# Gastrointestinal Effects and Tolerance of Nondigestible Carbohydrate Consumption

**DOI:** 10.1093/advances/nmac094

**Published:** 2022-08-30

**Authors:** Annemarie R Mysonhimer, Hannah D Holscher

**Affiliations:** Department of Food Science and Human Nutrition, University of Illinois, Urbana, IL, USA; Department of Food Science and Human Nutrition, University of Illinois, Urbana, IL, USA; Division of Nutritional Sciences, University of Illinois, Urbana, IL, USA

**Keywords:** dietary fiber, nonstarch polysaccharides, resistant starch, laxation, transit time, bloating, flatulence, gastrointestinal function

## Abstract

Nondigestible carbohydrates (NDCs) are food components, including nonstarch polysaccharides and resistant starches. Many NDCs are classified as dietary fibers by the US FDA. Because of their beneficial effects on human health and product development, NDCs are widely used in the food supply. Although there are dietary intake recommendations for total dietary fiber, there are no such recommendations for individual NDCs. NDCs are heterogeneous in their chemical composition and physicochemical properties—characteristics that contribute to their tolerable intake levels. Guidance on tolerable intake levels of different NDCs is needed because overconsumption can lead to undesirable gastrointestinal side effects, further widening the gap between actual and suggested fiber intake levels. In this review, we synthesize the literature on gastrointestinal effects of NDCs that the FDA accepts as dietary fibers (β-glucan, pectin, arabinoxylan, guar gum, alginate, psyllium husk, inulin, fructooligosaccharides and oligofructose, galactooligosaccharides, polydextrose, cellulose, soy fiber, resistant maltodextrin/dextrin) and present tolerable intake dose recommendations for their consumption. We summarized the findings from 103 clinical trials in adults without gastrointestinal disease who reported gastrointestinal effects, including tolerance (e.g., bloating, flatulence, borborygmi/rumbling) and function (e.g., transit time, stool frequency, stool consistency). These studies provided doses ranging from 0.75–160 g/d and lasted for durations ranging from a single-meal tolerance test to 28 wk. Tolerance was NDC specific; thus, recommendations ranged from 3.75 g/d for alginate to 25 g/d for soy fiber. Future studies should address gaps in the literature by testing a wider range of NDC doses and consumption forms (solid compared with liquid). Furthermore, future investigations should also adopt a standard protocol to examine tolerance and functional outcomes across studies consistently.

## Teaser

**Statement of Significance**: This review synthesizes the findings from clinical trials that assessed gastrointestinal tolerance to nondigestible carbohydrates that qualify as dietary fibers and provides recommendations for tolerable intake levels. These recommendations will inform new food product testing and help to address the fiber gap in the general population.

## Introduction

Nondigestible carbohydrates (NDCs) are food components, including nonstarch polysaccharides and resistant starches, that cannot be broken down by human small intestinal enzymes (1). The US FDA defined dietary fiber as “non-digestible soluble and insoluble carbohydrates (with three or more monomeric units), and lignin that are intrinsic and intact in plants; isolated or synthetic non-digestible carbohydrates (with three or more monomeric units) determined to have physiological effects that are beneficial to human health” (2). Dietary fibers are heterogeneous, varying in their chemical composition, linkages, and degrees of polymerization (chain length); these factors also affect the physicochemical properties of the fibers and thus their health benefits (3, 4). Therefore, it is important to have broad categorical dietary fiber intake recommendations and resources available that provide tolerable intake recommendations for specific NDCs to help assist with further design and testing of products containing NDCs.

Consumption of NDCs provides health benefits such as reducing blood glucose, cholesterol, blood pressure, and energy intake; improving laxation; and increasing intestinal mineral absorption (5). NDCs that the FDA has approved as dietary fibers include β-glucan, psyllium husk, cellulose, guar gum, locust bean gum, pectin, hydroxypropylmethylcellulose, inulin, fructooligosaccharides (FOS), soy fiber, polydextrose, rice bran, sugar cane fiber, cross-linked phosphorylated resistant starch 4, galactooligosaccharides (GOS), resistant maltodextrin/dextrin, glucomannan, arabinoxylan, alginate, and mixed plant cell wall fibers (contain ≥2 of the following: cellulose, pectin, lignin, β-glucan, arabinoxylan) (2, 5, 6).

**FIGURE 1:**
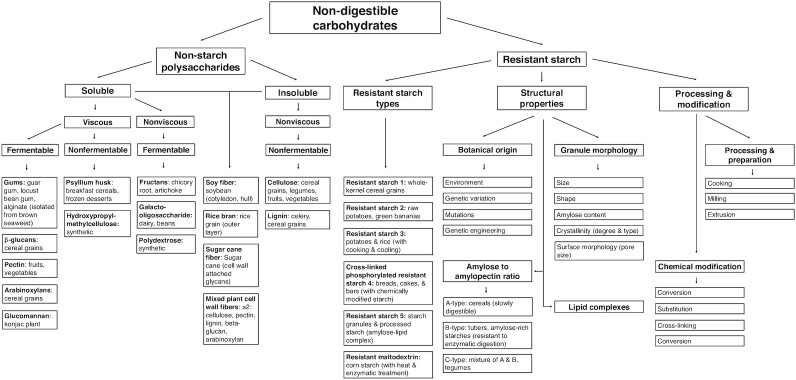
Nondigestible carbohydrate (NDC) classifications. NDCs can be classified into nonstarch polysaccharides and resistant starch. Nonstarch polysaccharides can be further classified by physicochemical properties of solubility, viscosity, and fermentability, whereas resistant starch can be further grouped by resistant starch type (e.g., resistant starch 1, resistant starch 2), structural properties (e.g., botanical origin, granule morphology), processing, and modification.

NDCs can be classified as nonstarch polysaccharides or resistant starches. Nonstarch polysaccharides can be further classified by their degree of solubility, viscosity, and fermentability, whereas resistant starches can be further classified by type, structural properties, and processing and modification (**Figure 1**). Many NDCs occur naturally in fruit, vegetables, legumes, and whole grains (7). However, because of their beneficial effects on health and food functional properties, NDCs are also isolated or synthesized and added to products such as bakery products, cereals, beverages, spreads, and desserts (1, 8, 9). Indeed, the market process of reformulation is defined as altering products to decrease nonnutritional properties (e.g., fat, calories, sugar) while also often enriching beneficial nutrients like fiber (10).

Although adequate intake recommendations have been established for total dietary fiber (14 g/1000 kcal) (11), no such intakes have been determined for individual or total NDCs. The importance of providing additional consumption guidelines for various NDCs is underscored by the fact that few people meet the adequate intake for total dietary fiber. Indeed, according to the 2020–2025 Dietary Guidelines for Americans report, only 10% and 3% of adult women and men, respectively, consume adequate fiber daily (11). Notably, recent work has reported that reformulation to enrich or fortify fibers in food products would help to narrow the gap between actual and recommended intake levels (10, 12). However, as overconsumption of certain NDCs can lead to undesirable side effects, such as bloating and flatulence, that reduce consumer acceptance, additional research and recommendations on the tolerable upper limits of NDC consumption stand to benefit consumers. Ultimately, to better incorporate NDCs into the diet and narrow the fiber gap, it is important to understand the gastrointestinal effects, including both tolerance and function, of consuming different NDCs.

**FIGURE 2:**
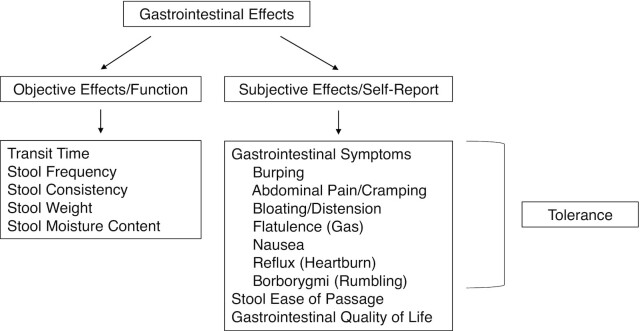
Interrelationship between gastrointestinal effects and tolerance. Gastrointestinal effects encompass both objective effects or function and subjective effects or self-reported measures. Objective effects include measurements of gastrointestinal function such as transit time, stool frequency, and stool consistency. Subjective effects include experience of gastrointestinal symptoms, stool ease of passage, and gastrointestinal quality of life. Self-reporting of gastrointestinal symptoms, such as bloating and flatulence, is defined as tolerance.

Gastrointestinal effects are physiologic responses to the consumption of foods, beverages, or supplements. Scientists have used numerous methods to measure these effects during clinical trials, such as requesting that participants complete subjective records or visual analog scales about digestive symptoms (e.g., bloating, flatulence, cramping). Objective measurements, such as tracking intestinal transit time (e.g., radio-opaque markers, food dye) or collecting stool samples to analyze in the laboratory (e.g., weight, moisture content, consistency), are also frequently used. Because of the many methods used to measure gastrointestinal effects, it is important that these techniques be categorized to support standard protocols and terminology across research studies (13), particularly in considering the interrelationship between gastrointestinal effects and tolerance (**Figure 2**).

Gastrointestinal effects encompass objective effects or function and subjective effects or self-reported measures (1, 8). Objective effects include measurements of gastrointestinal function such as transit time, stool frequency, and stool consistency. Subjective effects include the individual's reported experience of gastrointestinal symptoms, stool ease of passage, and gastrointestinal quality of life. Last, although tolerance falls within the subjective gastrointestinal effects category, its definition is also more exclusive. Tolerance is the self-reporting of various gastrointestinal symptoms, including burping, abdominal pain/cramping, bloating/distension, flatulence (gas), nausea, reflux (heartburn), and borborygmi (rumbling). Functional properties of NDCs, such as fermentability, can affect tolerance. Indeed, the extent of fiber fermentation (i.e., whether intestinal microorganisms fully or partially ferment the fiber) may affect the acceptability of consuming a product containing an NDC (1), underscoring the importance of in vitro testing of fermentability (14) and other functional properties of different fibers (e.g., solubility, viscosity) to complement clinical research.

It is also important to note that there is interindividual variation in digestion and health effects from any dietary consumption. Diet, intestinal microbiota composition, genetics, and health status are some of the factors that may contribute to these interindividual variations (15–17). Although general conclusions about normal compared with abnormal characteristics of bowel movements can be made, there are currently only definitions of abnormal conditions, including diarrhea and constipation. Functional diarrhea is defined as loose or watery stools, without predominant abdominal pain or bothersome bloating, occurring in more than 25% of stools (18). Functional constipation is defined as having ≥2 of the following: *1*) straining in >25% of defecations, *2*) lumpy or hard stools >25% of defecations, *3*) sensation of incomplete evacuation >25% of defecations, *4*) sensation of anorectal obstruction/blockage >25% of defecations, *5*) manual maneuvers to facilitate >25% of defecations, and *6*) <3 spontaneous bowel movements per week (19). Healthy bowel movements fall within the spectrum between diarrhea and constipation and are perceived by the individual as acceptable. Individuals should also experience no more than mild symptoms like bloating, flatulence, and borborygmi. Ultimately, it is important to consider interindividual variation in gastrointestinal effects and make conservative recommendations for the upper limits of tolerable intake of specific NDCs.

Herein, we aimed to *1*) synthesize the literature on gastrointestinal effects of NDCs classified as dietary fibers in adults without gastrointestinal disease and *2*) present tolerable intake dose recommendations for NDC consumption that are likely to result in no more than minimal intolerance symptoms in adults without gastrointestinal disease.

## Methods

This review provides an updated summary of recent literature on the gastrointestinal effects of consuming NDCs that meet the FDA definition of dietary fiber. As we aimed to provide an update to the most recent review on this topic, which was published by Grabitske and Slavin in 2009 (1), we included the studies they had summarized. We also included articles in the Diet-Related Fibers and Human Health Outcomes Database, which at the time of our search included articles through 2019 (20) that reported outcomes related to tolerance (e.g., burping, cramping/pain, bloating, flatulence, nausea, reflux, borborygmi), as well as functional effects such as fecal bulk, laxation, and transit time. In addition, we summarized relevant studies reported in the FDA's 2018 “Review of the Scientific Evidence on the Physiological Effects of Certain Non-Digestible Carbohydrates” (21) that included gastrointestinal effects. Last, PubMed was used to capture additional clinical trials published from 2019 through 2022 that were beyond the scope of these resources that reported on NDCs classified as dietary fibers and reported gastrointestinal effects. Within PubMed, we searched for each NDC using the following terms: alginate, guar gum, psyllium husk, inulin, inulin-type fructans, fructooligosaccharides, oligofructose, polydextrose, galactooligosaccharides, soy fiber, resistant maltodextrin/resistant dextrin/soluble corn fiber, β-glucan, pectin, arabinoxylan, and cellulose. Studies in healthy adults who had any reporting of tolerance or gastrointestinal effects, including primary and secondary outcomes and adverse effects, were included in this review. Trials that studied participants with gastrointestinal disease were excluded. Findings of gastrointestinal effects, including both tolerance and functional effects, and dose recommendations are summarized below for each NDC. In total, we reviewed the findings from 103 clinical trials that provided doses ranging from 0.75–160 g/d and lasted for durations ranging from a single-meal tolerance test on the short end all the way up to 28 wk on the long end.

### Current status of knowledge

#### β-Glucan

β-Glucans are soluble, viscous, fermentable (22) nonstarch polysaccharides with (1,3), (1,4), or (1,6) glycosidic bonds linking D-glucose units found intrinsic and intact within cereal cell walls (23). β-Glucans can bind bile acids, and consumption of ≥3 g/d has been shown to reduce blood cholesterol concentrations and the risk of coronary heart disease (21, 24, 25). Isolated β-glucan added to foods can serve as a stabilizing agent through its structure and water-binding capacity (26). **Table 1** displays the 5 studies, 2 acute (single test meal) and 3 chronic (4–8 wk), on β-glucans that reported tolerance measures for doses between 3 and 28 g/d (22, 27–30).

**TABLE 1:** Clinical trials that studied β-glucan consumption in adults without gastrointestinal disease^1^

Study	Population	Design	Duration	Dose	Control (vehicle)	Treatment (vehicle)	Assessment	Responses
Wolever, 2021 (27)	Adults in Canada (*n* = 191; 119 F, 72 M) with LDL cholesterol 3–5 mmol/L, 43.3 ± 14.3 y, BMI 29.7 ± 5.2	Randomized, blinded, parallel	4 wk	3 g/d	Rice powder (water)	Oat β-glucan (water)	Gastrointestinal symptoms	Flatulence ↑ from baseline with both treatment and control at 2 wk.^2^ Diarrhea ↑ from baseline at both 2 and 4 wk with treatment and control, with prevalence being higher with treatment at 2 wk.^2^ Constipation ↑ in control compared with treatment at 2 wk.^2^ Distension ↑ from baseline with control at 2 wk.^2^ Abdominal pain ↑ from baseline with both treatment and control at 2 wk but remained only with control at 4 wk.^2^
Morales, 2021 (28)	Adults in Spain (*n* = 52; 38 F, 14 M) with HCL, 18–65 y, BMI 18.5 to <30	Randomized, controlled, double blind, parallel	8 wk	3.5 g/d	Maltodextrin (commercial asparagus and zucchini creams and gazpacho)	β-Glucan from Glucanfeed S.L. powdered shiitake mushrooms (commercial asparagus and zucchini creams and gazpacho)	Adverse events, gastrointestinal symptoms	*n* = 3 dropped out due to diarrhea with treatment. Adverse events with treatment were swelling (*n* = 4), heartburn (*n* = 3), and flatulence (*n* = 2). Adverse events with control were swelling (*n* = 3), flatulence (*n* = 2), and diarrhea (*n* = 2)
Queenan, 2007 (22)	Adults in the United States (*n* = 75; 50 F, 15 M) with HCL, 22–65 y, BMI ≤30	Randomized, double blind, parallel	6 wk	6 g/d	Dextrose (beverage)	β-Glucan, 12 g 54% pure oat bran concentrate (beverage)	Survey rating stool frequency, consistency, bloating, flatulence	Treatment ↑ flatulence, but significance was not reported
Dicks, 2022 (29)	Adults in Germany (*n* = 22; 14 F, 8 M) with impaired glucose tolerance; F: 42.7 ± 17.4 y, BMI 35.4 ± 6.3; M: 47.6 ± 18.2 y, BMI 31.8 ± 5.9	Randomized, controlled, double blind, acute crossover	4 h, ≥1-wk washout	8.1 g/d	None (smoothie and soup)	β-Glucan from BIO Pleurotus powdered oyster mushrooms (smoothie and soup)	Adverse events (nausea, headache, flatulence)	No differences in adverse events between treatments
Hakkola, 2021 (30)	Healthy adults in Finland (*n* = 14; 7 F, 7 M), 18–64 y, BMI 18.5–30	Randomized, double blind, postprandial, 3-period crossover	48-h run-in and study day, 24- to 48-h follow-up, 2-wk washouts	28 g/d	None (low-phenolic, low-fiber meals)	Treated Fazer Mills oat bran concentrate with high (>1000 kDa), medium (524 kDa), and low (82 kDa) molecular weight β-glucan (powder + low-phenolic, low-fiber meals)	Diaries (5 d before and after study day) on abdominal pain, cramping, bloating, flatulence, diarrhea, constipation, and other (3-point scale)	Gut well-being was similar between the different molecular weight β-glucan meals. The background diet caused discomfort, and the most reported symptoms were flatulence and constipation

1 BMI is presented as kg/m^2^. HCL, hypercholesterolemia.

2 Differences were statistically significant (*P* ≤ 0.05).

Mild flatulence was reported in most of these studies. The study providing 28 g/d reported no differences in gut well-being between β-glucans of differing molecular weights (30). However, in another study providing 3.5 g/d, 3 participants dropped out of the study due to diarrhea (28). One possible explanation for these differing effects is the food form of the fibers—although the higher dose was administered as part of a meal, the lower amount was provided in a liquid state within creams and gazpacho. Therefore, when consumed in a solid food vehicle, this NDC appears to be well tolerated up to 28 g/d β-glucan, which would include the ≥3-g/d therapeutic dose to reduce the risk of coronary heart disease. However, more studies should be conducted at varying doses and in different food forms to confirm these findings and provide more context to recommend a tolerable consumption level.

#### Pectin

Pectin is a soluble, viscous, fermentable nonstarch polysaccharide with covalently linked galacturonic acids found in plant cell walls (31). Consumption of ≥9 g/d pectin helps to reduce blood cholesterol concentrations (21, 24, 32). From a food science perspective, isolated pectin added to foods is valued for its gel-forming capacity (32). **Table 2** summarizes 3 pectin studies that reported tolerance outcomes—these studies provided 6 to ∼38.5 g/d pectin to participants over 5–9 wk (33–35).

**TABLE 2:** Clinical trials that studied pectin consumption in adults without gastrointestinal disease

Study	Population	Design	Duration	Dose	Control (vehicle)	Treatment (vehicle)	Assessment	Responses
Spiller, 1980 (33)	Healthy adults in the United States (*n* = 42), 23–60 y, ±20% ideal body weight	2-wk low-residue diet, 3-wk treatment	5 wk	6 g/d	Sucrose (low-residue diet)	Sunkist Growers pectin (low-residue diet)	Transit time, fecal weight	Fecal weight ↓ 0.32 g/d with pectin and 15 g/d with control from wk 2 to 5.^1^
Cummings, 1979 (34)	Healthy males in the United Kingdom, (*n* = 5), 21–24 y	Single group	9 wk on a controlled diet, with pectin added during the last 6 wk	36 g/d	None (controlled diet)	Pectin (controlled diet)	Bowel habit, transit time	Pectin ↑ stool weight slightly at wk 6 and 9 compared with the control week.^1^
Fleming, 1983 (35)	Healthy males in the United States (*n* = 5), 21–32 y, normal weight	Metabolic study	63 d (7 periods, 9 d each)	∼38.5 g/d	None (fiber-free diet)	Sigma Chemical Co. pectin (fiber-free diet)	Flatulence, transit time, stool frequency and output	Pectin ↓ transit time and ↑ flatulence compared with the basal diet

1 Differences were statistically significant (*P* ≤ 0.05).

In these 3 pectin studies, mostly functional effects were reported (e.g., stool weight), with only the highest dose mentioning other tolerance symptoms (e.g., flatulence). Therefore, future studies should be conducted explicitly measuring various gastrointestinal symptoms, as well as function, so that a recommendation can be made regarding an upper intake level of pectin that is well tolerated. This will also be important to determine whether the therapeutic dose of ≥9 g/d pectin to reduce blood cholesterol would be well tolerated.

#### Arabinoxylan

Arabinoxylans are soluble, viscous, fermentable nonstarch polysaccharides with β-xylopyranosyl sugars linked by (1,4) bonds with terminal L-arabinofuranosyl substituted at positions 2 and 3 that are found in the cell walls of cereal grains (36, 37). Consumption of 2.6–15 g/d arabinoxylan has been demonstrated to attenuate blood glucose and insulin concentrations (21). Isolated arabinoxylan is important for its gel-forming capacity in foods (38). **Table 3** displays the 2 studies, 1 acute (single test meal) and 1 chronic (10 wk), that reported functional gastrointestinal effects, ranging in dose from 9.4–15.1 g/d arabinoxylan (39, 40).

**TABLE 3:** Clinical trials that studied arabinoxylan consumption in adults without gastrointestinal disease^1^

Study	Population	Design	Duration	Dose	Control (vehicle)	Treatment (vehicle)	Assessment	Responses
Scarpellini, 2018 (39)	Healthy adults in Belgium (*n* = 13; 7 F, 6 M), 18–42 y, BMI 21.6 ± 1.6	Single blind, crossover, placebo controlled	12 h prior to study and next morning for test	9.4 g/d	Maltodextrin (warm water)	Fugeia NV's Brana Vita 200 AXOS from wheat bran extract (warm water)	Transit time	Treatment did not affect transit time compared with control
Lu, 2004 (40)	Adults in Australia (*n* = 15; 9 F, 6 M) with T2D, 60 ± 2 y, BMI 28.1 ± 0.9	Randomized, crossover	Two 5-wk periods	15.1 g/d	50% whole wheat, 50% white flour (bread and muffins)	14% AX, 50% whole wheat, 36% white flour (bread and muffins)	Scored daily stool frequency from 1 (much less) to 9 (much greater than usual); side effects, fecal weight, 24-h fecal collection	Treatment ↑ stool frequency (*P* < 0.05) and wet weight (*P* = 0.05) compared with control.^2^ No differences in flatulence, distension, or cramping between treatment and control

1 BMI is presented as kg/m^2^. AX, arabinoxylan; AXOS, arabinoxylooligosaccharide; T2D, type 2 diabetes.

2 Differences were statistically significant (*P* ≤ 0.05).

Although 9.4 g/d mixed with warm water did not affect transit time, 15.1 g/d added to bread and muffins increased fecal wet weight. The many differences between these 2 studies make direct comparisons difficult. First, supplementing fibers in different forms (i.e., liquids compared with solids) can affect tolerance (41). Furthermore, both the dose and the intervention duration varied. Although 9.4 g/d arabinoxylan was consumed 12 h before the study and on the morning of the test, 15.1 g/d was consumed for a 5-wk period, demonstrating the importance of longer-term consumption for gastrointestinal benefits.

Ultimately, few studies measured gastrointestinal effects and tolerance of arabinoxylan consumption. Thus, additional studies are necessary to provide a recommendation on a tolerable intake level for arabinoxylan. This will be important to determine the tolerability of therapeutic doses of arabinoxylan to reduce blood glucose and insulin concentrations.

#### Guar gum

Guar gum is a soluble, viscous, fermentable nonstarch polysaccharide classified as a galactomannan, which has a linear structure of (1,4)-linked mannose and side-chain (1,6)-linked galactose sugars (42). Guar gum is isolated from the guar bean endosperm (43). Consumption of ≥15 g/d guar gum has been shown to reduce blood cholesterol concentrations (21, 24, 44). When added to foods, it acts as an emulsifier and thickener (21). **Table 4** displays the 7 chronic (2-wk to 3-mo) guar gum consumption studies that reported gastrointestinal effects, ranging in dose from 5–40 g/d (41, 45–50).

**TABLE 4:** Clinical trials that studied guar gum consumption in adults without gastrointestinal disease^1^

Study	Population	Design	Duration	Dose	Control (vehicle)	Treatment (vehicle)	Assessment	Responses
Yasukawa, 2019 (45)	Healthy adults in Japan (*n* = 44; 22 F, 22 M) with a tendency toward diarrhea, 20–49 y, BMI 22.4 ± 2.5	Randomized, double blind, placebo controlled, parallel	3 months	5 g/d	Maltodextrin (sachet)	Sunfiber PHGG (sachet)	Stool consistency and frequency, gastrointestinal symptoms	PHGG improved stool consistency,^2^ but not frequency, compared with control. No differences in gastrointestinal symptoms between or within groups
Penagini, 1986 (46)	Healthy adults in Italy (*n* = 6)	Crossover	6 wk	11.4 g/d	None (controlled diet, free diet)	Guar gum (controlled diet)	Stool weight, frequency, transit time	Stool weight, frequency, and transit time did not differ between treatments
Jenkins, 1980 (41)	Patients in the United Kingdom (*n* = 11; 7 F, 4 M) with hyperlipidemia, 56 ± 3 y, 116 ± 6% desirable weight	2-wk study, 6-wk follow-up	8 wk	13 g/d	Cholestyramine (medication)	Guar gum (crispbread, hydrated, semihydrated)	Constipation, distension, flatulence, stool looseness	Medication ↑ constipation and abdominal distention in *n* = 3. Hydrated and semihydrated guar ↑ excessive flatulence and stool looseness. Guar crispbread ↑ mild flatulence and improved constipation in *n* = 3
Cummings, 1978 (48)	Healthy males in the United Kingdom (*n* = 19); 20–38 y (*n* = 3 consumed guar)	Crossover	9 wk (3-wk basal diet, two 3–wk treatments)	∼20 g/d	None (controlled basal diet)	Hercules Powder Co. guar gum (controlled basal diet)	Transit time (radio-opaque pellets), fecal weight	Guar gum diluted marker in stool compared with baseline,^2^ indicating ↑ stool frequency
Alam, 1998 (49)	Healthy males in Switzerland (*n* = 10; 28–41 y), normal body weight	Double-blind, randomized, crossover	3 wk (two 1-wk treatments, one 1-wk washout)	31 g/d (3000-kcal diet), 21 g/d (2000-kcal diet)	None (liquid formula diet)	Benefiber PHGG (liquid formula diet)	Questionnaire to assess stool frequency and consistency, defecation ease, flatulence, and abdominal pain	PHGG ↑ number of normal consistency stools compared with control.^2^
Takahashi, 1993 (50)	Healthy males in Japan (*n* = 8), 22–38 y	4-wk controlled diet, 4-wk free diet, 4-wk treatment	12 wk	36 g/d	None (controlled and free diet periods)	Sunfiber PHGG (controlled diet)	Stool weight and frequency	PHGG ↑ stool frequency in weeks 3 and 4 and stool weight compared with controlled and free diet.^2^
Pasman, 1997 (study 1) (47)	Females in the Netherlands (*n* = 17) with obesity, 38.5 ± 2.3 y, BMI 32.2 ± 0.9	Crossover	2 wk (1-wk treatments)	40 g/d	None (orange juice)	Benefiber PHGG (orange juice)	Recorded complaints (tolerance not directly assessed)	Guar gum caused *n* = 2 to report flatulence

1 BMI is presented as kg/m^2^. PHGG, partially hydrolyzed guar gum; T2D, type 2 diabetes.

2 Differences were statistically significant (*P* ≤ 0.05).

The most frequently assessed outcomes following guar gum consumption were stool frequency and consistency, with 4 of the 7 studies reporting improvements in 1 or both of these measures. The studies showed that consuming 5–11.4 g/d guar gum did not result in undesirable tolerance effects. Consumption of 13–40 g/d guar gum generally resulted in mild to moderate flatulence. However, consumption of 15 g/d hydrated (powder mixed in soup, milk, or juice) and semihydrated (half of the fiber dose mixed in liquid and half in bread) guar gum resulted in excessive flatulence (41). Interestingly, 10–18 g/d guar gum (mean: 13 g/d) incorporated into crispbread led to only mild flatulence (41).

In summary, studies that included 5–40 g/d guar gum demonstrated that guar gum consumption is well tolerated up to 11.4 g/d. However, if higher doses are desired for therapeutic purposes, incorporating guar gum into solid foods (e.g., crispbread) instead of liquid formulations is recommended. Therefore, a therapeutic dose of ≥15 g/d guar gum to reduce cholesterol would be mostly well tolerated. With a little higher dose needed than 11.4 g/d, guar gum would still likely produce only mild gastrointestinal effects if provided in a solid form.

#### Alginate

Alginate is a soluble, viscous, fermentable polyuronic nonstarch polysaccharide extracted from brown seaweed cell walls (51). Consumption of 1.5–8 g/d alginate has been demonstrated to lower postprandial blood glucose concentrations (6). When isolated alginate is added to foods, it helps to improve texture through its gelling and stabilizing capacity (21). **Table 5** reports 4 alginate studies that included gastrointestinal effects, ranging in dose from 3–45 g/d and duration from a single-meal test to 12 wk (52–55).

**TABLE 5:** Clinical trials that studied alginate consumption in adults without gastrointestinal disease^1^

Study	Population	Design	Duration	Dose	Control (vehicle)	Treatment (vehicle)	Assessment	Responses
Nam, 2022 (55)	Patients in Korea (*n* = 210; 72 F, 138 M) without significant disease undergoing EGD, 20–80 y	Open label, randomized, controlled, parallel arm	3 d	3 g/d	None (none)	Taejoon Pharm Co. Lamina-G sodium alginate, 1 g 3×/d (not specified)	Gastrointestinal symptoms (abdominal pain, epigastric pain/soreness, heartburn, acid reflux, nausea/vomiting, borborygmi, abdominal distension, belching)	Epigastric pain/soreness ↑ with control, but ↓ with treatment.^2^ Acid regurgitation and epigastric soreness, belching, and borborygmi ↓ during follow-up with treatment compared with baseline.^2^
Wolf, 2002 (52)	Healthy adults in the United States (*n* = 30; 19 F, 11 M), 36 ± 2 y, BMI 21–28	Randomized, double blind, placebo controlled, crossover	2 OGTTs	3.75 g/d	Gum arabic + guar gum (glucose beverage)	Kelco sodium alginate (glucose beverage)	Questionnaire to report severity of nausea, cramping, distention, vomiting, burping, and reflux from 0 (absent) to 3 (severe)	No difference in tolerance symptoms between treatments
Torsdottir, 1991 (53)	Males in Sweden (*n* = 7) with T2D, 39–58 y, BMI 20–30	Ingestion on 2 d, randomized	3 h on each of 2 test days	5 g/d	None (control meal)	Drammen Protanal L-60, Protan A-S sodium alginate, 75% soluble fiber (control meal)	Gastric emptying rate	Treatment resulted in slower gastric emptying compared with control.^2^
Jensen, 2012 (*AJCN*) (54)	Adults in Denmark (*n* = 96) with obesity, 20–55 y, BMI 30–45	Parallel, double blind, placebo controlled	12 wk	45 g/d	Placebo (energy-restricted diet + preload)	FMC Biopolymers Protanal LFR 5/60 sodium alginate (energy-restricted diet + preload)	VAS to rate heartburn, reflux, nausea, distension, abdominal pain, constipation, flatulence, and diarrhea; reporting of adverse events graded as mild, moderate, or severe	Mean maximum VAS scores ↑ with alginate compared with control at wk 6.^2^

1 BMI is presented as kg/m^2^. *AJCN, American Journal of Clinical Nutrition*; EGD, esophagogastroduodenoscopy; OGTT, oral glucose tolerance test; T2D, type 2 diabetes; VAS, visual analog scale.

2 Differences were statistically significant (*P* ≤ 0.05).

The most frequently assessed outcome for alginate was self-reported tolerance symptoms. Participants who consumed 3.75 g/d alginate for 3 d did not report intolerance, but consuming 45 g/d alginate for 12 wk increased tolerance symptom scores. The acute study (3 h on 2 test days) that used an intermediate dose of 5 g/d alginate only measured gastric emptying rate, which was slowed by alginate consumption.

Therefore, the evidence from these 4 studies testing 3–45 g/d suggests alginate is well tolerated up to 3.75 g/d. Although this dose can likely be higher, additional studies are needed to determine acceptable doses intermediary to a dose of 45 g/d as this amount contributed to increased gastrointestinal symptoms. Therefore, a therapeutic dose of 1.5–8 g/d to improve postprandial blood glucose would be well tolerated at least up to 3.75 g/d.

#### Psyllium husk

Psyllium (ispaghula) husk is a soluble, viscous, nonfermentable nonstarch polysaccharide isolated from the seed husk of the *Plantago ovata* Forsk plant (56, 57). Mucilage from the psyllium seed is made up of 75% xylose and 23% arabinose (56). Consumption of ≥7 g/d psyllium husk has been found to benefit health by decreasing coronary heart disease risk (21, 24, 26). From a food product development perspective, isolated psyllium husk is added to foods for its water-holding capacity (58). **Table 6** summarizes 4 chronic (∼2–6.5 wk) studies and 1 single-meal psyllium husk consumption study that reported gastrointestinal effects, ranging in dose from 9–30 g/d (59–63).

**TABLE 6:** Clinical trials that studied psyllium husk consumption in adults without gastrointestinal disease^1^

Study	Population	Design	Duration	Dose	Control (vehicle)	Treatment (vehicle)	Assessment	Responses
Cherbut, 1994 (61)	Healthy males in France (*n* = 6), 21–32 y	4-period crossover	Participants studied on 4 occasions, separated by 7 d	15 g/d	None (glucose solution)	Ispaghul Trouette–Perret ispaghula husk (glucose solution)	Duodenojejunal motor activity	Ispaghula ↑ propagation length and velocity of activity compared with control.^2^
Marlett, 2000 (59)	Healthy adults in the United States (*n* = 15; 7 F, 8 M), 18–30 y, BMI 24.2 ± 0.9	2-period crossover	Two 7-d periods	15 g/d	None (controlled diet)	Smooth Texture Metamucil psyllium seed husk (controlled diet)	Stool viscosity, moisture, and weight	Psyllium ↑ stool viscosity, daily wet weight, moisture, and frequency compared with control.^2^
Marteau, 1994 (62)	Healthy adults in France (*n* = 7; 2 F, 5 M), 21–35 y	2-period crossover	Two 15-d periods	18 g/d	Sucrose (pellets, low-fiber controlled diet)	Spagulax ispaghula husk (pellets, low-fiber controlled diet)	Transit time, stool weight, flatulence	Ispaghula ↑ stool frequency and wet weight compared with control.^2^
Jalanka, 2019 (63)	Healthy adults in the United Kingdom (*n* = 7), 18–65 y, BMI 18–30	Randomized, controlled, double blind, 3-period crossover	6-d treatments, 1-wk washouts	10.5, 21 g/d	Maltodextrin (water)	Metamucil psyllium (water)	Gastrointestinal transit, fecal water content	Psyllium ↑ stool water content of participants
Eherer, 1993 (60)	Healthy adults in the United States (*n* = 9; 2 F, 7 M), 20–36 y	4-d experimental diarrhea protocol repeated on 4 occasions with ≥10 d between episodes; additional dose–response study	∼46 d (*n* = 6 for additional dose–response study ∼28 d)	18 g/d; dose–response: 9, 18, 30 g/d	None (empty gelatin capsules + water)	Lafayette Pharmaceutical Co. Konsyl psyllium (capsules + water)	Stool consistency and viscosity	Psyllium ↑ stool viscosity and weight compared with control.^2^ Doses of 9, 18, and 30 g/d psyllium caused near linear ↑ in viscosity

1 BMI is presented as kg/m^2^. T2D, type 2 diabetes.

2 Differences were statistically significant (*P* ≤ 0.05).

The most frequently assessed outcomes for psyllium husk were stool properties, with 4 of the 5 studies reporting improvements. In addition, at 18 g/d (pellets with a low-fiber controlled diet), tolerance symptoms did not differ compared with the control. Therefore, doses up to 18 g/d or higher may be well tolerated when eaten with solid foods. However, more studies should be conducted explicitly measuring tolerance symptoms such as flatulence and bloating with various doses and food forms (e.g., solid or liquid) to fully evaluate appropriate intake levels. In summary, from studies testing consumption of 9–30 g/d, we recommend a conservative tolerable intake dose of up to 15 g/d psyllium husk. Therefore, a therapeutic dose of ≥7 g/d psyllium husk to reduce risk of coronary heart disease also falls within this range to be well tolerated.

#### Inulin-type fructans

Fructans are soluble, nonviscous, fermentable nonstarch polysaccharides consisting of fructose monomers linked to a terminal sucrose molecule (64). Inulin-type fructans include inulin, FOS, and oligofructose (OF). These fibers provide health benefits, including improved laxation, insulin sensitivity, intestinal barrier function, satiety, and mineral absorption, as well as reduced blood triglyceride concentrations (65). Although inulin is a long-chain fructan with a degree of polymerization ≥10, FOS is a short-chain fructan with a degree of polymerization of 3–9 (64), and OF is a plant short-chain fructan with a degree of polymerization of 2–20 (66). The fructose units of inulin-type fructans are typically connected via β-(2,1) bonds (67). Fructans are found in wheat, onions, bananas, garlic, leeks, asparagus, chicory, oats, soybeans, and artichokes (68, 69). Fructans with a linear structure and β-(2,1) bonds are classified as inulin (69). Chicory inulin specifically has a degree of polymerization of 2–60, with an average of 12 (69). We found 34 inulin-type fructan studies that reported gastrointestinal effects and separated them into 17 inulin studies and 17 FOS/OF studies.

#### Inulin

Inulin contributes to health by improving laxation at doses ranging from 15–50 g/d (6). Isolated inulin is added to foods as a bulking agent (21), to contribute to mouth feel, and to allow for reduction of fat in products. **Table 7** displays 17 chronic (3–20 wk) studies testing inulin doses ranging from 0.75–50 g/d (70–86).

**TABLE 7:** Clinical trials that studied inulin consumption in adults without gastrointestinal disease^1^

Study	Population	Design	Duration	Dose	Control (vehicle)	Treatment (vehicle)	Assessment	Responses
Nishimura, 2015 (70)	Healthy adults in Japan (*n* = 47; 39 F, 8 M), 33–70 y, BMI 22 ± 3	Randomized, double blind, placebo controlled	4 wk	0.75 g/d	None (barley tea with 10% coffee)	Chicory root extract from Tenshin Farm chicory (beverage)	VAS questionnaire on fecal properties	No change in fecal VAS scores
Ramnani, 2010 (71)	Healthy adults in the United Kingdom (*n* = 66; 33 F, 33 M) 18–50 y, BMI 20–30	3-arm parallel, placebo controlled, double blind	2-wk run-in, 3-wk treatment, 3-wk washout	5 g/d	None (water-based beverages with sugar and fruit/vegetable flavors provided by Unilever)	Inulin (2 types of fruit/vegetable juice shots provided by Unilever)	Daily diaries recording stool frequency and consistency, abdominal pain, bloating, and flatulence	Mild ↑ flatulence with one of the inulin treatments compared with control.^2^
Holscher, 2014 (79)	Healthy adults in the United States (*n* = 29), 20–36 y, BMI 20–29	Randomized, double blind, placebo controlled, 3-period crossover	Three 21-day periods, 1-wk washouts	5, 7.5 g/d	None (chocolate chews)	Ingredion BioAgave agave inulin (chocolate chews)	Tolerance assessed with daily and weekly questionnaires; 3 fecal samples collected on days 16–20 each period	Inulin ↑ intolerance scores (scores low) and mild bloating, flatulence, and rumbling frequency compared with control.^2^ Abdominal pain and rumbling intensity ↑ with 7.5 g/d inulin compared with control. 7.5 g/d inulin ↑ stool frequency and softened stool.^2^
Ripoll, 2010 (80)	Healthy adults in France (*n* = 53; 19 F, 34 M), 18–67 y, mean BMI 22.05	*1*) Double blind, crossover; *2*) randomized, double blind	*1*) Three 6-d periods; *2*) 4 wk	*1*) 5, 7.8 g/d; *2*) 5 g/d	Sucrose (coffee drink)	Inulin from inulin-rich soluble chicory extract (coffee drink)	Flatulence, bloating, abdominal pain, stool consistency, stool number	*1*) 7.8 g/d inulin ↑ mild abdominal discomfort after 1 wk compared with control.^2^*2*) No difference in gastrointestinal symptoms. Short- and long-term consumption of 5 g/d inulin was well tolerated
Kolida, 2007 (81)	Healthy adults in the United Kingdom (*n* = 30; 15 F, 15 M), 19–35 y	Double blind, placebo controlled, crossover	Three 2-wk treatments, two 1-wk washouts	5, 8 g/d	Maltodextrin (chocolate drink)	Sensus Frutafit IQ inulin (chocolate drink)	Daily diaries to record stool frequency and consistency; abdominal pain, bloating, and flatulence rated 0 (none) to 3 (severe)	5 g/d inulin ↑ stool number, bloating, and flatulence compared with the washout period.^2^
Costabile, 2010 (82)	Healthy adults in the United Kingdom (*n* = 32; 18 F, 14 M), 20–42 y, BMI 20–30	Double-blind, randomized, crossover	Two 3-wk study periods, 3-wk washout period	10 g/d	Maltodextrin (not specified)	Bayer BioScience GmbH very long-chain inulin (not specified)	Stool frequency and consistency, abdominal pain, bloating, flatulence	Inulin ↑ mild and moderate bloating compared with control.^2^
Russo, 2008 (83)	Healthy males in Italy (*n* = 15), 18.8 ± 0.7 y, BMI 20–25	Randomized, double blind, crossover	2-wk run-in, two 5-wk study periods, 8-wk washout	11 g/d	None (pasta with 100% durum wheat semolina)	Orafti Raftiline HP-Gel inulin (pasta with 11% inulin, 86% semolina, 3% durum wheat vital gluten)	Questionnaire on gastrointestinal symptoms	No side effects with inulin (flatulence, bloating, postprandial fullness) or change in bowel habit
Sairanen, 2007 (84)	Healthy adults in Finland (*n* = 66; 44 F, 22 M), 22–60 y, BMI 19–40	Randomized, 3-way parallel group, double blind	33 d, 12-d baseline and 3-wk intervention	12 g/d	None (fermented milk)	Orafti Raftiline HP inulin (fermented milk with probiotics)	Record of defecation frequency and gastrointestinal symptoms; collection of all feces; intestinal transit time and stool weight	Fermented milk with probiotics + inulin ↑ gastrointestinal symptoms, particularly flatulence, compared with control.^2^
Grasten, 2003 (85)	Healthy adults in Finland (*n* = 14; 11 F, 3 M), 36.1 ± 13.3 y, BMI 22.7 ± 3.5	Parallel group	3 wk	13 g/d	None (run-in phase)	Orafti Raftiline inulin (wheat bread)	Daily questionnaire with stool frequency and consistency (5-point scale); intestinal symptoms of flatulence, stomach pain, diarrhea, and other (4-point scale)	Mild and moderate flatulence was most common symptom
Dahl, 2005 (86)	Adults in Canada (*n* = 15) with wheelchairs, 23–57 y, BMI 14.3–28.9	Double blind, crossover	3 wk	13 g/d	None (starch-thickened beverage)	Sensus Frutafit IQ inulin (starch-thickened beverage)	Chart records to determine stool frequency and laxative administration; output size to determine weighted stool frequency; interviews to monitor beverage acceptability and tolerance	No gastrointestinal discomfort or change in stool frequency, but inulin ↑ weighted frequency compared with control.^2^
Pedersen, 1997 (72)	Healthy females in Denmark (*n* = 64), 20–36 y, BMI 21.9 ± 2.6	Randomized, double blind, crossover	Two 4-wk periods	14 g/d	None (low-fat spread)	Orafti Raftiline inulin (low-fat spread)	Gastrointestinal symptoms assessed with questionnaires	Inulin ↑ discomfort from flatulence and other symptoms compared with control.^2^ No intestinal adaptation to this dose of inulin
Gibson, 1995 (73)	Healthy adults in the United Kingdom (*n* = 8; 1 F, 7 M), 21–48 y, BMI 18.7–25.4)	2 controlled feeding studies	45 d	15 g/d	Sucrose (free-form and biscuits)	Orafti Raftiline inulin (free-form and biscuits)	Daily diary to record times of radiopaque markers taken, stools passed, and events like diarrhea and flatulence	No significant differences
Van Dokkum, 1999 (74)	Healthy males in the Netherlands (*n* = 12), 23 ± 3 y, mean BMI 23	Randomized, double blind, diet controlled	Four 3-wk treatments	15 g/d	None (orange juice + controlled basal diet)	Inulin (orange juice + controlled basal diet)	Stool weight, intestinal transit	Fecal parameters did not differ
Causey, 2000 (75)	Males in the United States (*n* = 12) with hypercholesteremia, 27–49 y, BMI ≤32	Randomized, double blind, crossover	Two 3-wk treatments	20 g/d	Sucrose (ice cream + controlled diet)	Inulin (ice cream + controlled diet)	Transit time, stool weight, symptom evaluations	Inulin ↑ complaints of flatulence during early consumption, but flatulence disappeared within 4 d
Slavin, 2011 (76)	Healthy males in the United States (*n* = 12), 27–49 y, BMI ≤32	Randomized, double blind, crossover	3 wk per treatment	20 g/d	Corn syrup (ice cream + controlled diet)	Sensus Frutafit chicory inulin (ice cream + controlled diet)	Stool weight, intestinal transit time, stool frequency and consistency	Inulin ↑ flatulence compared with control.^2^
Kruse, 1999 (77)	Healthy adults in Germany (*n* = 11; 5 F, 6 M), 26–53 y, BMI 22.8 ± 4.4 (F) and 25.8 ± 1.6 (*n* = 8 completed study)	Crossover	8-d run-in control diet, 64-d intervention	22–34 g/d	None (control diet: 45% fat, 40% carb, 15% protein)	Cosucra Fibruline inulin (diet: 30% fat, 55% carb, 15% protein)	Symptoms of nausea, vomiting, belching, acid reflux, bloating, flatulence, diarrhea, rumbling, and cramps rated 0 (absent) to 3 (severe)	Inulin ↑ mild to moderate flatulence and bloating compared with control.^2^*n* = 2 dropped out due to gastrointestinal discomfort
Castiglia-Delavaud, 1998 (78)	Healthy males in France (*n* = 9), 21.5 ± 2.5 y, BMI ≤25	3-period crossover	28-d treatments (gradual ↑ to full dose)	50 g/d	None (control diet)	Agro-industries chicory inulin (control diet)	Stool frequency and weight	Inulin caused diarrhea in *n* = 1. Inulin ↑ wet and dry stool weight and frequency compared with control.^2^

1 BMI is presented as kg/m^2^. VAS, visual analogue scale.

2 Differences were statistically significant (*P* ≤ 0.05).

The most assessed outcome for inulin was stool frequency, which was improved when participants consumed inulin in 5 of the reviewed studies. However, flatulence and bloating were reported in 9 and 4 of the studies, respectively. Low doses of 0.75–5 g/d inulin led to only mild flatulence, medium doses of 7.5–20 g/d led to mild and moderate symptoms, and higher doses (≥22 g/d) resulted in study dropouts.

Considering the 17 studies of inulin testing doses of 0.75–50 g/d, we recommend a dose of inulin up to 5 g/d to avoid more than mild flatulence. However, doses up to 20 g/d can be tolerated well by some individuals, aside from mild to moderate bloating and flatulence. Therefore, although a therapeutic dose up to 15–20 g/d inulin to improve laxation would likely result in some intolerance symptoms, they would likely be only mild or moderate symptoms.

#### Fructooligosaccharides and oligofructose

Compared with inulin, both FOS and OF have shorter chain lengths, necessitating study of these NDCs separately in terms of gastrointestinal effects. FOS and OF at 10–15 g/d have been shown to improve mineral absorption (6). In addition, FOS and OF added to foods contribute to sweetness and can help reduce the amount of sugar in products (65). **Table 8** displays 17 FOS and OF studies testing doses ranging from 4–160 g/d and duration from 1–16 wk (73, 74, 87–101).

**TABLE 8:** Clinical trials that studied fructooligosaccharide and oligofructose consumption in adults without gastrointestinal disease^1^

Study	Population	Design	Duration	Dose	Control (vehicle)	Treatment (vehicle)	Assessment	Responses
Buddington, 1996 (89)	Healthy adults in the United States (*n* = 12; 6 F, 6 M), 20–34 y (*n* = 1 removed from analysis due to constipation)	Controlled diet 42 d + 4 g/d FOS days 7–32	42 d (FOS days 7–32)	4 g/d	None (controlled diet)	FOS (tablets and flavored drink mix, controlled diet)	Daily log to record changes in stool volume, consistency, and frequency; flatulence; stool consistency	FOS softened stool at the beginning of consumption, and after treatment ended, stools became even less formed. Flatulence was reported, but mostly only with beginning of controlled diet
Swanson, 2002 (96)	Healthy adults in the United States (*n* = 68; 42 F, 26 M), ≥18 y	Randomized, double blind, placebo controlled, parallel group	4-wk baseline, 4-wk treatment	6 g/d	Sucrose (cornstarch + noncarbonated beverage)	GTC Nutrition NutraFlora FOS (cornstarch + noncarbonated beverage)	7-d bowel function forms, fresh stool sample collection at weeks 4, 6, and 8	No difference in stool frequency
Geyer, 2008 (100)	Healthy adults in Switzerland (*n* = 16; 8 F, 8 M), 18–57 y, normal BMI	Placebo controlled, double blind, crossover	2-wk crossover, 2-wk washout	6.4 g/d	Molasses (syrup)	Yacon, 32% FOS (syrup)	Transit time; stool frequency and consistency; side effects assessed using questionnaires	FOS ↓ transit time compared with control.^2^
Venema, 2005 (94)	Healthy adults in the Netherlands (*n* = 30; 18 F, 12 M)	Randomized, placebo controlled, double blind, 5-period crossover	Five 2-wk treatments	7.8 g/d	Sucrose (raspberry jam)	Cerestar Actilight 950P FOS (raspberry jam)	Adverse events determined with well-being questionnaire; symptom questionnaire (excess flatus, stomach rumbling, burping, bloating, abdominal pain, abdominal cramps, nausea, and vomiting); daily stool consistency and frequency	FOS ↑ stools of thin/very thin consistency (observation) and stool frequency at 2 wk^2^ compared with control
Cummings, 2001 (87)	Healthy adults in the United Kingdom (*n* = 244; 113 F, 131 M), 18–75 y	Randomized, double blind, placebo controlled	5 wk (preliminary week, 2-wk preholiday, 2-wk holiday)	10 g/d FOS	Maltodextrin (water)	FOS (water)	Stool number, size, and consistency; record amount and consistency of stools; discomfort, symptoms (fever, abdominal pain, vomiting, abnormal bloating, chronic flatulence, diarrhea, constipation)	FOS ↑ stool frequency before the holiday and well-being and flatulence during the holiday compared with control.^2^
Goetze, 2008 (99)	Healthy adults in Switzerland (*n* = 20; 12 F, 8 M), 20–37 y, BMI 20.1–25.3	Double blind, randomized, crossover	18 wk	10 g/d	Maltodextrin (beverage of choice)	Orafti Raftilose P95 FOS (beverage of choice)	Abdominal symptoms and general well-being; rectal sensations assessed by VAS	FOS ↑ bloating and ↓ general well-being compared with run-in with maltodextrin.^2^
Alles, 1996 (92)	Healthy males in the Netherlands (*n* = 24), 19–28 y, BMI 21.7 ± 1.9	3-period crossover	5 wk (7 d/treatment, two 7-d washouts)	5, 15 g/d	Glucose (water)	Orafti Raftilose P95 FOS (water)	Ba-impregnated rings swallowed daily and counted in fecal samples; time of defecation; stool form; complaints of flatulence, bloating, abdominal pains or cramps, eructation, nausea, vomiting, and stomach pain or cramps	15 g/d FOS ↑ flatulence compared with control.^2^
Andermann, 2021 (93)	Patients (*n* = 15) undergoing reduced-intensity allo-HCT and adult controls (*n* = 16) in the United States, >18 y	Phase I pilot, single arm, dose escalation	3 wk	5, 10, 15 g/d	None (none)	Cosucra Fibrulose F97 FOS (powder dissolved in food or beverage)	Tolerability	FOS was well tolerated at 10 g/d without significant adverse effects
Gibson, 1995 (73)	Healthy adults in the United Kingdom (*n* = 8; 1 F, 7 M), 21–48 y, BMI 18.7–25.4	2 controlled feeding studies	45 d	15 g/d	Sucrose (free-form and biscuits)	Orafti Raftilose OF (free-form and biscuits)	Daily diary to record times of radiopaque markers consumed, stools passed, and events like diarrhea and flatulence	OF ↑ stool frequency compared with control
Van Dokkum, 1999 (74)	Healthy males in the Netherlands (*n* = 12), 23 ± 3 y, mean BMI 23	Randomized, double blind, diet controlled	Four 3-wk treatments	15 g/d	None (orange juice + controlled basal diet)	FOS (orange juice + controlled basal diet)	Stool weight, intestinal transit	Fecal parameters did not differ
Pol, 2018 (98)	Adults in the Netherlands (*n* = 55; 36 F, 19 M) who are overweight or with obesity, 20–60 y, BMI 25–35	Parallel, triple blind, placebo controlled	12 wk	16 g/d	High-maltose maize syrup, maize syrup, and starch (chocolate chip granola bars)	Sensus Frutalose L92 OF (chocolate chip granola bars)	Liking of the bars and tolerance: bloating, regurgitation, flatulence, nausea, and looser stools	In weeks 1 and 2, OF ↑ flatulence and bloating compared with baseline.^2^
Ten Bruggencate, 2006 (95)	Healthy males in the Netherlands (*n* = 34), 27.7 ± 1.7 y, BMI 23.2 ± 0.5	Double blind, placebo controlled, crossover	6 wk (two 2-wk treatments, 2-wk washout)	20 g/d	Sucrose (lemonade)	Orafti Raftilose P95 FOS, 93% pure (lemonade)	Scored gastrointestinal symptoms with VAS and collected stool samples	FOS ↑ fecal wet weight, flatulence, bloating, and mucin excretion (indicating mucosal irritation) compared with control.^2^
Scholtens, 2006 (97)	Healthy adults in the Netherlands (*n* = 121; 6 F, 6 M), 18–35 y, mean BMI 20.7	Randomized, double blind, placebo controlled, crossover	2 wk	25–30 g/d	Maltodextrin (sachets)	Orafti Raftilose P95 FOS (sachets)	Diary with questions on stool frequency and consistency and gastrointestinal symptoms (constipation, diarrhea, flatulence, bloating, stomachache, cramps, nausea, regurgitations, vomiting)	FOS ↑ stool frequency and flatulence compared with control.^2^
Francois, 2014 (101)	Healthy adults in Belgium (*n* = 20; 10 F, 10 M), 46.9 ± 15.9 y, BMI 24.4 ± 3.4	Placebo controlled, 3-period crossover	2-wk run-in, three 2-wk treatments, 2-wk washouts	Week 1: 15 g/d, week 2: 30 g/d	None (noncarbonated soft drinks)	Sensus Frutalose L92 OF (noncarbonated soft drinks)	72-h stool samples to analyze fecal output and moisture; questionnaires scoring frequency and severity of 18 gastrointestinal symptoms; overall symptom measure	OF ↑ overall gastrointestinal symptoms 2-fold with 15 g/d and 1.9-fold with 30 g/d compared with control.^2^ 30 g/d OF ↑ stool moisture compared with control.^2^
Bouhnik, 1999 (91)	Healthy adults in France (*n* = 40; 22 F, 18 M), 18–47 y	Randomized, 5-way parallel group	7 d	5, 10, 20, 40 g/d	Saccharose (powder)	Actilight FOS (powder)	Daily chart to rate tolerance symptoms (excess flatus, borborygmi, bloating, abdominal pain) from 0 (no symptoms) to 3 (severe); stool frequency and consistency	40 g/d FOS ↑ excess flatulence compared with lower doses and control.^2^
Briet, 1995 (90)	Healthy adults in France (*n* = 14)	Dose–response	FOS ingested throughout day occasionally (period 1: 1×/wk) or regularly (period 2: every day)	Doses ↑ until diarrhea and/or symptom graded 3 (severe) occurred or when subjects did not want to continue	None (candies)	FOS (candies)	Flatulence, borborygmi, bloating, abdominal cramping, diarrhea	Excessive flatulence with >30 g/d FOS, borborygmi and bloating with >40 g/d, and abdominal cramps and diarrhea with 50 g/d
Clausen, 1998 (88)	Healthy adults in Denmark (*n* = 12; 8 F, 4 M), 27–56 y	Crossover	3 d for each of 5 dose levels, 1-wk washouts	20, 40, 80, 160 g/d	Lactulose (beverage)	Ferrosan A/S Idolax OF, 95.7% pure (beverage)	24-h stool volume (collected day 3 of each treatment dose)	Lactulose and FOS both had a laxative effect, but lactulose ↑ fecal volume twice as high as FOS.^2^ Due to diarrhea, *n* = 4 could not complete the final dose of lactulose

1 BMI is presented as kg/m^2^. allo-HCT, allogeneic hematopoietic cell transplantation; FOS, fructooligosaccharide; OF, oligofructose; VAS, visual analog scale.

2 Differences were statistically significant (*P* ≤ 0.05).

The most frequently assessed outcome following FOS and OF consumption was stool frequency, with 4 studies that provided 6.4, 10, 15, and 25–30 g/d specifically reporting improved stool frequency. However, 7 studies that provided 10–40 g/d also reported flatulence from FOS or OF consumption. Although low to medium doses of 10–20 g/d seemed to lead to mild flatulence, >30 g/d led to excessive flatulence. One study was particularly helpful in determining tolerance of different dose levels by providing increasing doses of FOS. This research reported that >30 g/d FOS resulted in excessive flatulence, >40 g/d FOS led to borborygmi and bloating, and >50 g/d resulted in diarrhea and abdominal cramping (90).

The wide variety of the 17 studies on FOS and OF, ranging in dose from 4–160 g/d, demonstrates the importance of dose when consuming specific NDCs. For instance, although healthy adults appear to be able to consume up to 30 g/d before experiencing excessive flatulence, most consumers (particularly those with digestive conditions) would not want to consume this high of a dose. Therefore, a reasonable recommendation to avoid intolerance symptoms is 7.8 g/d FOS or OF. However, to achieve a therapeutic dose of 10–15 g/d to improve mineral absorption, FOS and OF would still be well tolerated, with likely only mild flatulence.

#### Galactooligosaccharides

GOS is a soluble, nonviscous, fermentable nonstarch, complex collection of oligosaccharides often created from lactose molecules using β-galactosidases (102). Connected to a terminal glucose, 1–8 sugars of galactose are bonded by β-(1,1), β-(1,2), β-(1,3), β-(1,4), or β-(1,6) linkages (102). GOS has been shown to provide the health benefit of improving intestinal calcium absorption with a dose of 20 g/d (6), and isolated GOS is added to foods as a bulking agent and to improve texture (21). **Table 9** displays the 5 chronic (2–16 wk) GOS studies that reported gastrointestinal effects, ranging in dose from 2.5–20 g/d (103–107).

**TABLE 9:** Clinical trials that studied galactooligosaccharide consumption in adults without gastrointestinal disease^1^

Study	Population	Design	Duration	Dose	Control (vehicle)	Treatment (vehicle)	Assessment	Responses
Schaafsma, 2021 (105)	Healthy adults in the Netherlands (*n* = 69), 30–50 y, BMI 19.5–25	Double blind randomized, placebo controlled, crossover	9 wk	5.2 g/d	Skim milk powder (powder mixed in water)	Biotis GOS dairy test product (powder mixed in water)	Tolerability (flatulence, nausea, bloating)	No differences in tolerability
Walton, 2012 (103)	Healthy adults in the United Kingdom (*n* = 37; 21 F, 16 M), 50–81 y, BMI 19.7–38.4	Randomized, double blind, placebo controlled, crossover	3-wk treatments, 3-wk washouts	8 g/d	None (orange juice)	Friesland Campina Vivinal GOS (orange juice)	Stool frequency	No difference in stool frequency between treatments
Ito, 1990 (107)	Healthy males in Japan (*n* = 12), 26–48 y	Single blind, crossover	7 d/period	2.5, 5, 10 g/d	None (apple juice)	Oligomate-50 GOS (apple juice)	Stool weight, frequency, diarrhea	10 g/d GOS did not change stool weight and frequency or lead to diarrhea compared with control
Davis, 2010 (106)	Healthy adults in the United States (*n* = 18; 5 F, 13 M), 19–50 y	Single-blind	16 wk (2-wk baseline, each dose 3 wk, 2-wk washout)	2.5, 5, 10 g/d	None (chocolate chews)	GTC Nutrition Purimune GOS (chocolate chews)	Bowel movements, stool consistency, discomfort, flatulence, abdominal pain, bloating	No difference in stool frequency or tolerance symptoms
Van den Heuvel, 2000 (104)	Females in the Netherlands (*n* = 12) in postmenopause, 55–65 y, BMI 20.7–32.4	Double blind, randomized, crossover	Two 9-d treatments, 19-d washout	20 g/d	Sucrose (yogurt drink)	Borculo Domo Ingredients Elix'or T-GOS (yogurt drink)	Reported gastrointestinal adverse effects	20 g/d T-GOS did not lead to any adverse side effects (gastrointestinal complaints or stool changes) compared with control

1 BMI is presented as kg/m^2^. B-GOS, bimuno-galactooligosaccharides; GOS, galactooligosaccharides; T-GOS, transgalactooligosaccharides.

The most commonly assessed outcome for GOS was stool frequency—this outcome remained unchanged in all the studies reviewed. Undesirable tolerance effects were also unchanged in the studies. Therefore, up to 20 g/d GOS appears to be well tolerated, which is the dose that has also been demonstrated to improve calcium absorption.

#### Polydextrose

Polydextrose is a soluble, nonviscous, fermentable nonstarch polysaccharide with a highly branched structure, a degree of polymerization of 2–120, and a mean degree of polymerization of 12 (108). This fiber consists of glucose monomers linked by α- and β-(1,2), (1,3), (1,4), and (1,6) glycosidic bonds, with (1,6) being the most common (108). Polydextrose provides the health benefit of reducing energy intake at doses ranging from 12–25 g/d (6), and isolated polydextrose is added to foods for its bulking effect, texture, and keeping moisture in foods (21). **Table 10** displays 11 polydextrose studies, 5 acute (single meal) and 6 chronic (4–11 wk), that reported gastrointestinal effects, ranging in dose from 4–56.7 g/d (109–119).

**TABLE 10:** Clinical trials that studied polydextrose consumption in adults without gastrointestinal disease^1^

Study	Population	Design	Duration	Dose	Control (vehicle)	Treatment (vehicle)	Assessment	Responses
Kondo, 1996 (109)	Healthy adults in Japan (*n* = 5; 3 F, 2 M), 22–48 y	Postprandial	6-h monitoring, each beverage on separate day	7 g/d	10.2 g/d lactulose (soft drink)	PDX (Nakakita Pharmaceuticals Sapitus 5289 soft drink)	Orocecal transit time, flatulence (no quantitative assessment)	Orocecal transit time was constant for all treatments and flatulence was common
Costabile, 2012 (110)	Healthy adults in the United Kingdom (*n* = 31; 16 F, 15 M), 18–50 y, BMI 19–25	Placebo controlled, double blind, crossover	2-wk lead-in, 3-wk treatments, 3-wk washout	8 g/d	Maltodextrin (powder)	Danisco UK Litesse Ultra PDX (powder)	Bowel habits, quality of life	PDX ↓ abdominal discomfort and pain compared with control.^2^
Monsivais, 2011 (112)	Healthy adults in the United States (*n* = 36; 22 F, 14 M), 20–34 y, BMI 18–30	Double blind, preload	6 testing days over 6 wk, ≥1 wk between sessions	11.8 g preload provided twice	Isoenergetic, low-fiber preload and lower-energy, low-fiber preload (snack + beverage preload)	Sta-Lite III PDX (snack + beverage preload)	Computerized VAS to rate nausea at 20- min intervals	Nausea did not differ between preloads
Jie, 2000 (113)	Healthy adults in China (*n* = 120; 54 F, 66 M), 32.9 y (M) and 29.4 y (F)	Placebo controlled, randomized, double blind, 4-group parallel arm	28 d	4, 8, 12 g/d	0 g/d PDX (blinded, not specified)	Danisco Litesse PDX (warm water)	Bowel function (frequency and ease of defecation)	PDX improved frequency and ease of defecation compared with control.^2^
Hull, 2012 (114)	Healthy adults in the United Kingdom (*n* = 34; 24 F, 10 M), ≥18 y, BMI 18.5–25	Randomized, single blind, placebo controlled, 3-period crossover	Over 3 wk, each participant visited test facility on 3 occasions (1-wk washout between each visit)	6.25, 12.5 g/d	Glucose (strawberry flavor drinking yogurt)	DuPont Litesse PDX (strawberry flavor drinking yogurt)	Questions on liking drinks and discomfort symptoms (bloating, nausea, headache) using VAS	6.25 g/d PDX ↑ bloating compared with control and 12.5 g/d PDX.^2^ 6.25 g/d PDX ↓ belching compared with control.^2^
Timm, 2013 (115)	Healthy adults in the United States (*n* = 36; 18 F, 18 M), ≥18 y, BMI 18.5–30	Randomized, double blind, placebo controlled, crossover	10-d treatments, 2-wk washout	20 g/d	None (muffin and cereal)	Tate and Lyle STA-LITE PDX (muffin and cereal)	Fecal samples, gastrointestinal tolerance questionnaires	PDX ↑ fecal wet weight, frequency, flatulence, and borborygmi and softened stool compared with control.^2^
Vester Boler, 2011 (116)	Healthy males in the United States (*n* = 21), 21–28 y, BMI 20–34	Crossover	21-d periods	21 g/d	None (snack bar)	Danisco Litesse II PDX (snack bar)	Fecal samples, gastrointestinal symptoms	PDX ↑ flatulence compared with control.^2^ All tolerance scores were low (only slight discomfort)
Astbury, 2013 (117)	Healthy adults in the United Kingdom (*n* = 21; 12 M, 9F), 18–45 y, BMI 19–25	Randomized, within subject, crossover	4 visits, ≥7 d between visits	6.3, 12.5, 25 g/d	Maltodextrin (chocolate-flavored liquid preload)	Danisco Litesse Ultra PDX (chocolate-flavored liquid preload)	Questionnaire on abnormal gastrointestinal symptoms or discomfort (e.g., bloating, nausea, loose bowel movement, flatulence) in 24 h after leaving lab	No reports of gastrointestinal distress
King, 2005 (118)	Healthy adults in the United Kingdom (*n* = 15; 8 F, 7 M), mean 30.1 y, mean BMI 22.7	4-period crossover, repeated measures	Four 10-d periods	12.5, 25 g/d	Sucrose (yogurt)	Danisco Litesse PDX (yogurt)	Recorded experiences of bloating and nausea with VAS on days 1 and 10 of each period	No differences in bloating or nausea between treatments
Achour, 1994 (119)	Healthy males in France (*n* = 7), 27 ± 2 y, within 10% ideal body weight	Control phase, acute ingestion, chronic ingestion	38 d	30 g/d	None (controlled diet)	Pfizer PDX (fruit juice)	Transit time, abdominal symptoms	PDX did not change transit time or ↑ abdominal symptoms compared with control
Konings, 2014 (111)	Adults in the Netherlands (*n* = 18; 9 F, 9 M) who were overweight, 20–50 y, BMI 27.2 ± 0.4 (F) and 27.3 ± 0.5 (M)	Single blind, randomized, crossover	4 treatment sessions, 1-wk washouts	56.7 g/d	None (control diets)	Sta-lite PDX (control diet)	Well-being determined with questionnaire on symptoms of nausea, abdominal bloating, stomach-intestinal cramps, diarrhea, and other	No nausea, burping, headache, vomiting, or dizziness. Diarrhea was present in 1 female and 1 male with PDX

1 BMI is presented as kg/m^2^. PDX, polydextrose; VAS, visual analog scale.

2 Differences were statistically significant (*P* ≤ 0.05).

The most frequently assessed outcome from polydextrose consumption was stool frequency, with 2 studies reporting improved frequency with polydextrose consumption. Polydextrose was well tolerated up to 56.7 g/d in most participants; however, this dose led to diarrhea in 2 participants. Others have recommended an intake of 4–12 g/d polydextrose to have beneficial effects without causing gastrointestinal distress (108, 113).

Considering previous recommendations and these 11 studies testing doses of 4–56.7 g/d, consumption of up to 12 g/d polydextrose is recommended to limit intolerance symptoms, with doses up to 30 g/d contributing to only mild symptoms. Therefore, a therapeutic dose of 12–25 g/d to reduce energy intake falls within this range of only limited to mild symptoms.

#### Cellulose

Native cellulose is an abundant polymer found in plant cells walls. Various derivatives are used as functional additives in food products. The current review is focused on cellulose derivatives (i.e., an isolated fiber) as trials that used intrinsic and intact fibers in whole foods were not included. Cellulose derivatives are insoluble, nonviscous, and nonfermentable, and they comprise tightly packed linear (1,4)-linked D-glucopyranose chains (120). They provide the health benefit of improved laxation at ≥14 g/d (21, 24) and can serve as an emulsifier, film-forming agent, fat replacer, and encapsulator, among other properties (121). **Table 11** displays the 2 chronic (5–9 wk) cellulose studies that reported gastrointestinal effects, ranging in dose from ∼14–38.5 g/d (33, 35).

**TABLE 11:** Clinical trials that studied cellulose consumption in adults without gastrointestinal disease

Study	Population	Design	Duration	Dose	Control (vehicle)	Treatment (vehicle)	Assessment	Responses
Spiller, 1980 (33)	Healthy adults in the United States (*n* = 42), 23–60 y, within 20% ideal body weight	Low-residue diet 2 wk, then 3-wk low-residue diet + treatment	5 wk	14 g/d	Sucrose (low-residue diet)	Brown and Co. Solka-floc cellulose (low-residue diet)	Transit time, fecal weight	Transit time ↓ by 2.5 d and fecal weight ↑ by 34 g/d with cellulose compared with baseline.^1^
Fleming, 1983 (35)	Healthy males in the United States (*n* = 5), 21–32 y, normal weight	Metabolic study	63 d (7 periods, each 9 d)	∼38.5 g/d	None (basal fiber-free diet)	ICN Pharmaceuticals Inc. Alphacel cellulose (basal diet)	Flatulence; colonic function assessed by transit time, fecal frequency, output, and composition	Cellulose ↑ fecal output and frequent defecations

1 Differences were statistically significant (*P* ≤ 0.05).

Fecal weight or output was increased by cellulose consumption in 2 studies that provided 14–38.5 g/d. Thus, both doses had beneficial effects on laxation. However, more studies are needed on gastrointestinal effects specific to tolerance rather than just stool characteristics to make a specific tolerable intake recommendation. Ultimately, doses up to 38.5 g/d cellulose appear to be well tolerated and a reasonable recommendation dose, because cellulose is insoluble, nonviscous, and generally not fermentable. Therefore, it is unlikely to contribute to symptoms such as flatulence and bloating, while also providing the health benefit of laxation.

#### Soy fiber

Soy fiber is both soluble and insoluble and is isolated from the cotyledon and hull of soybeans (6, 122). The fiber from the cotyledon is mostly hemicellulose, whereas soy hull fiber is constituted of hemicellulose, cellulose, and pectin (6). Consumption of soy fiber can benefit health by attenuating blood cholesterol concentrations when doses of 10–26 g/d are consumed and improving laxation at 8.2–26 g/d (6). Isolated soy fiber is added to foods for its viscosity and water-holding capacity (123). **Table 12** displays the 6 soy fiber studies that assessed gastrointestinal effects, ranging in dose from 6–60 g/d and duration from a single-meal test to 28 wk (109, 122, 124–127). Of note, there many soy fiber derivatives. In the studies reviewed, the derivates included a soybean oligosaccharide formulation, never-dried soy pulp and purified soy fiber, soy polysaccharides, soy fiber, soybean hulls, and soy fiber.

**TABLE 12:** Clinical trials that studied soy fiber consumption in adults without gastrointestinal disease

Study	Population	Design	Duration	Dose	Control (vehicle)	Treatment (vehicle)	Assessment	Responses
Kondo, 1996 (109)	Healthy adults in Japan (*n* = 5; 3 F, 2 M), 22–48 y	Postprandial	6-h monitoring, each beverage on separate day	6 g/d	10.2 g/d lactulose (soft drink)	Soybean oligosaccharide (Calpis Food Engineering Oligo CC soft drink)	Orocecal transit time, flatulence (no quantitative assessment)	Orocecal transit time was constant across treatments and flatulence was common
Schweizer, 1983 (124)	Healthy adults in Switzerland (*n* = 6; 4 F, 2 M), 24–30 y, 102–110% ideal body weight	Crossover	2-wk control period, 3 wk/treatment	21 g/d	None (usual diet)	Nonpurified soy fiber, 39% (yogurt and soups), purified soy fiber, 79% (yogurt, milk, soup, or water)	Stool frequency and weight, transit time	Low and high soy fiber ↑ fecal wet weight by 19% and 38%, respectively, compared with control period.^1^
Tsai, 1983 (125)	Healthy males in the United States (*n* = 14), 20–30 y, 73.1 ± 5.3 (control) and 70.6 ± 5.9 (fiber)	Diet controlled, crossover	Two 17 d-feeding periods	25 g/d	None (low-fiber basal diet)	Soy polysaccharide (low-fiber basal diet)	Transit time, fecal moisture and dry matter, questionnaire determining fiber effect	Soy fiber ↑ total fecal wet weight and water content compared with control.^1^
Lo, 1986 (122)	Patients in the United States (*n* = 20) with hyperlipidemia, 27–70 y, 84–143% ideal body weight	Single blind, crossover	10-wk baseline diets, two 9-wk treatments	25 g/d	Starch (cookies)	Soy fiber (cookies)	Participants advised of possible side effects (abdominal bloating, flatus, eructation, ↑ stool bulk and urgency)	Soy fiber was well tolerated
Munoz, 1979 (126)	Healthy males in the United States (*n* = 10), 19–54 y	Crossover	30-d basal diet, 28–30 d/treatment (each fiber consumed by 4–6 males)	26 g/d	None (basal diet)	Soybean hulls, 87% (basal diet)	Fecal weight	Mean daily fecal weight ↑ with soybean hulls compared with baseline.^1^
Slavin, 1985 (127)	Healthy males in the United States (*n* = 16), 20–34 y	Randomized, 4-period crossover	4 treatments, 10 d each	30, 60 g/d	None (Ensure)	Soy fiber (Ensure and heat-treated Enrich)	Stool weight, transit time	Mean stool weights on Enrich + 30 g/d soy fiber, Ensure + 30 g/d, and Ensure + 60 g/d were 114.6, 100.2, and 150.3 g/d, respectively. Transit time was ↑ with Ensure alone (72.4 h) compared with fiber and self-selected diets (∼48 h).^1^ Soy fiber's effect on laxation was not affected by heat processing

1 Differences were statistically significant (*P* ≤ 0.05).

The most frequently reported outcome with soy fiber was stool weight. Study results revealed that consumption of soy fiber increased stool weight in 4 studies. Overall, gastrointestinal effects varied, likely related to fiber form. For example, 6 g/d soy fiber in soft drinks led to flatulence, yet when adults consumed 25 g/d in cookies, the soy fiber was well tolerated. Therefore, up to 25 g/d is recommended for preparations that incorporate soy fiber into a solid food vehicle, which also allows for therapeutic doses to improve blood cholesterol (10–26 g/d) and laxation (8.2–26 g/d).

#### Resistant maltodextrin/dextrin

Resistant maltodextrin/dextrin is a soluble, fermentable, nonviscous resistant starch also approved by the FDA as a dietary fiber (128). This category includes soluble corn fiber, resistant dextrin, resistant wheat dextrin, soluble wheat fiber, and wheat dextrin (21). These fibers are made up of glucose units linked by α-(1,6), α-(1,4), α-(1,3), and α-(1,2) glucosidic bonds and are isolated from partially hydrolyzed starches (21, 129). These carbohydrates can potentially span low-molecular-weight oligosaccharides to resistant maltodextrins. Therefore, it is important for researchers and food manufacturers to specify as much detail as possible when reporting. Only 1 study we reviewed on NUTRIOSE FB (Roquette Frères) purified dextrin reported the molecular weight of the NDC (130). There are several food company brand names associated with resistant maltodextrin. NUTRIOSE can be soluble corn, wheat, or pea fiber, and Fibersol (ADM/Matsutani, LLC) and Promitor (Tate & Lyle) both use resistant maltodextrin/soluble corn fiber. Resistant maltodextrin improves calcium absorption, retention, and bone formation at 10–20 g/d (6). Isolated resistant maltodextrin is added to foods because of its low viscosity, stability, and tasteless flavor profile (131). **Table 13** displays 11 studies, 1 acute (single meal) and 10 chronic (5–12 wk), testing resistant maltodextrin/dextrin in doses ranging from 7.5–100 g/d (112, 115, 116, 128, 130, 132–137).

**TABLE 13:** Clinical trials that studied resistant maltodextrin/dextrin consumption in adults without gastrointestinal disease^1^

Study	Population	Design	Duration	Dose	Control (vehicle)	Treatment (vehicle)	Assessment	Responses
Astina, 2022 (132)	*1*) Healthy adults in Thailand (*n* = 17), 18–55 y, BMI <23; *2*) adults in Thailand (*n* = 22) with fasting blood glucose <126 mg/dL, 18–55 y	*1*) Randomized, single-blind, crossover controlled trial; *2*) single-arm prospective study	*1*) Acute; *2*) 12 wk	*1*) 3.8, 7.7 g; *2*) 7.7 g/d	Tapioca maltodextrin (oral nutrition supplement powder mixed in water)	Tapioca resistant maltodextrin (oral nutrition supplement powder mixed in water)	Ratings of abdominal pain, bloating, nausea, vomiting, and flatulence; Bristol Stool Scale	Oral nutrition supplements were well tolerated
Kavyani, 2021 (137)	Patients in Iran (*n* = 36; 17 F, 19 M) with NAFLD, 20–50 y, BMI 25–35	Double blind, parallel arm	12 wk	10 g/d	Maltodextrin (flavorless white powder with lunch and dinner, 20 g CSO, calorie-restricted diet)	NUTRIOSE 06FM resistant dextrin (flavorless white powder with lunch and dinner, 20 g CSO, calorie-restricted diet)	Adverse events	No differences in adverse events between treatments
Monsivais, 2011 (112)	Healthy adults in the United States (*n* = 36; 22 F, 14 M), 20–34 y, BMI 18–30	Double blind, preload	6 test days over 6 wk, ≥1 wk between sessions	11.8 g (SCF) or 12 g (soluble fiber dextrin) twice daily	None (isoenergetic, low-fiber solid snack + liquid preload and lower-energy, low-fiber solid snack + liquid preload)	Soluble fiber dextrin or Promitor SCF 70 (solid snack + liquid preload)	Computerized VAS to rate nausea at 20-min intervals	Nausea did not differ between preloads
Stewart, 2010 (135)	Healthy adults in the United States (*n* = 20; 10 F, 10 M), 38 ± 4 y (F) and 32 ± 5 y (M), BMI 26.0 ± 1.4 (F) and 24.3 ± 0.7 (M)	Single blind, crossover	14-d treatments, 21-d washout	12 g/d	Maltodextrin (apple sauce)	Promitor resistant starch, soluble fiber dextrin, Promitor SCF (apple sauce)	Gastrointestinal symptom surveys, stool samples	Fiber treatments ↑ bloating, cramping, flatulence, stomach noises, and gastrointestinal score compared with control.^2^ (Mean symptom scores were low.)
Fastinger, 2008 (134)	Healthy adults in the United States (*n* = 38; 19 F, 19 M), 26.6 ± 4.5 (0 g/d), 28.2 ± 6.1 (7.5 g/d), and 26.7 ± 4.2 y (15 g/d), BMI 25.1 ± 3.0 (0 g/d), 23.5 ± 2.9 (7.5 g/d), and 25.4 ± 3.7 (15 g/d)	Randomized, double blind, placebo controlled, parallel group	7 wk: 2-wk baseline, 3-wk treatment, 2-wk washout	7.5, 15 g/d	Maltodextrin (packet + liquid)	Fibersol-2 RMD + maltodextrin, RMD (packet + liquid)	Daily stool records (time, consistency, ease of passage); ranked burping, cramping, distension/bloating, flatulence, nausea, reflux (heartburn), and vomiting (4-point scale)	Few differences in bowel function and tolerance. No severe effects from resistant maltodextrin or differences in stool consistency or ease of passage. Fecal scores and ease of passage ratings were within normal range. Tolerance symptoms were rated none or mild
Jakeman, 2016 (133)	Healthy females in the United States (*n* = 14) in postmenopause, 39.9–79.7 y, BMI 19.9–36.1	Randomized, crossover, double blind, dose–response	50 d	10, 20 g/d	Maltodextrin (fruit-flavored juice and muffin)	Promitor SCF 85 (fruit-flavored juice and muffin)	Abnormal symptoms commonly caused by ↑ fiber (abdominal pain, bloating, flatulence, diarrhea, stomach noises)	No difference in gastrointestinal distress severity between doses
Timm, 2013 (115)	Healthy adults in the United States (*n* = 36; 18 F, 18 M), ≥18 y, BMI 18.5–30	Randomized, double blind, placebo controlled, crossover	10-d treatments, 2-wk washout	20 g/d	None (muffin and cereal, <15 g/d fiber background diet)	Promitor SCF (muffin and cereal, <15 g/d fiber background diet)	Fecal samples, gastrointestinal tolerance questionnaires	SCF ↑ stool wet weight, flatulence, and borborygmi compared with control.^2^
Vester Boler, 2011 (116)	Healthy males in the United States (*n* = 21), 21–28 y, BMI 20–34	Crossover	21-d periods	21 g/d	None (snack bar)	Promitor SCF (snack bar)	Fecal samples, gastrointestinal symptoms	SCF ↑ flatulence and reflux compared with control.^2^ Tolerance scores were low (only slight discomfort)
Burns, 2018 (136)	Healthy adults in the United States (*n* = 49; 28 F, 21 M), 26.3 ± 6.8 y, BMI 24.4 ± 3.3	Double blind, controlled, crossover	3 wk/treatment	15, 25 g/d	Maltodextrin (packet + beverage of choice)	Fibersol-2 RMD + maltodextrin, RMD (packet + beverage of choice)	Stool wet weight	25 g/d resistant maltodextrin ↑ stool wet weight compared with baseline.^2^
Hashizume, 2012 (128)	Adults in Japan (*n* = 30) with metabolic syndrome, 61.2 ± 11.6 (control) and 60.1 ± 8.9 y (RMD), BMI 26.8 ± 2.9 (control) and 28.1 ± 2.3 (RMD)	Randomized, double blind, placebo controlled, parallel group	12 wk	27 g/d	None (unsweetened tea)	Fibersol-2 RMD (unsweetened tea)	Subjective abdominal symptoms	With treatment, *n* = 5 reported symptoms (flatulence in 5, abdominal sound in 1), and with control, *n* = 1 reported symptoms (flatulence)
Vermorel, 2004 (130)	Healthy males in France (*n* = 10), 23.6 ± 2.6 y, BMI 22.3 ± 1.6	Crossover and dose–response	Two 31-d periods, 4-wk washout	↑ doses 20–100 g/d	Dextrose (water)	NUTRIOSE FB purified dextrin (water)	Diary on occurrence and intensity of flatulence, intestinal gurgle, flatulence, abdominal pain, and diarrhea; stools collected during 5 d after end of adaptation to determine fecal output	Treatment ↑ stool wet weight^2^ and did not cause severe digestive symptoms, except excessive flatulence and mild abdominal pain with >50 g/d

1 BMI is presented as kg/m^2^. CSO, *Camelina sativa* oil; NAFLD, nonalcoholic fatty liver disease; RMD, resistant maltodextrin; SCF, soluble corn fiber; VAS, visual analog scale.

2 Differences were statistically significant (*P* ≤ 0.05).

The most frequently assessed outcome for resistant maltodextrin/dextrin was fecal weight or output, which increased with consumption in 3 studies. Gastrointestinal symptoms were reported in 6 studies. Doses up to 12 g/d were well tolerated, doses of 12–27 g/d led to mild or moderate symptoms, and doses >50 g/d resulted in excessive flatulence and mild abdominal pain. Thus, we recommend a tolerable intake dose of resistant maltodextrin/dextrin up to 12 g/d. Therefore, this dose would reach therapeutic levels to improve calcium absorption (10–20 g/d), and a slightly higher dose would lead to only mild or moderate symptoms.

#### Nondigestible carbohydrate mixtures

Seven studies tested combinations of different NDCs (**Table 14**) (138–144), particularly combinations of inulin-type fructans. Two of these studies included inulin + FOS or OF, 1 included OF-enriched inulin, 1 included a mixture of short- and long-chain fructans, and 1 included FOS + guar gum. Inulin + OF combinations have been shown to improve mineral absorption at 8–10 g/d (6). In addition, 2 studies that did not contain inulin-type fructans included a mixture of psyllium + lactulose and a mixture of guar gum + alginate.

**TABLE 14:** Clinical trials that studied consumption of nondigestible carbohydrate mixtures in adults without gastrointestinal disease^1^

Study	Population	Design	Duration	Dose	Control (vehicle)	Treatment (vehicle)	Assessment	Responses
Langlands, 2004 (141)	Patients in the United Kingdom (*n* = 29; 15 F, 14 M) from colonoscopy waiting lists, 31–81 y	Single group	2 wk	7.5 g/d OF + 7.5 g/d inulin (15 g/d total)	None (none)	Orafti Raftiline HP inulin + Raftilose P95 OF (not specified)	Daily diary of any side effects	OF + inulin was well tolerated, but also ↑ flatulence (mild to moderate in 10, severe in 4), bloating (mild to moderate in 7, severe in 2) and had a laxative effect (mild to moderate in 10, severe in 1)
Brighenti, 1999 (140)	Healthy males in Italy (*n* = 12), 23.3 ± 0.5 y, BMI 25.7 ± 1.2	Crossover	Three 4-wk periods	9 g/d	None (rice cereal + 2% milk)	Cosucra Fibruline Instant inulin + FOS (rice cereal + 2% milk)	Intestinal habits estimated using questionnaire; stool weight, ease of defecation, consistency, abdominal pain, and flatulence	Inulin + FOS ↑ flatulence occasionally but was well tolerated
Healey, 2018 (144)	Healthy adults in New Zealand (*n* = 34; 21 F, 13 M), 19–65 y, BMI 18.5–30	Randomized, double blind, placebo controlled, crossover	3 wk	16 g/d	Maltodextrin (powder + beverage of choice)	Orafti Synergy1 OF-enriched inulin (powder + beverage of choice)	Gastrointestinal symptoms (e.g., flatulence)	Treatment ↑ moderate symptoms, especially flatulence, compared with control.^2^
Rumessen, 1998 (143)	Healthy adults in Denmark (*n* = 10; 5 F, 5 M), 18–25 y, non obese	Single blind, crossover, randomized	Test days separated by ≥48 h within 2–6 mo	10–30 g/d	Lactulose, fructose, sorbitol, sorbitol + glucose (water)	Orafti Raftilose FASC short-chain fructans, Raftiline FALC long-chain fructans (water)	Flatulence, distension, borborygmi, abdominal pain, diarrhea, and nausea (none, 0; mild, 1; moderate, 2; or severe, 3) at fixed (0.5-h) intervals for 7 h after ingestion (14 times); total symptom score	Treatment ↑ abdominal symptoms with increasing dose and decreasing chain length. Overall gastrointestinal effects similar to control
Tuohy, 2001 (139)	Healthy adults in the United Kingdom (*n* = 31; 17 F, 14 M), 18–50 y, BMI 20–30	2-period crossover	Two 21-d periods	6.6 g/d FOS, 3.4 g/d PHGG	None (biscuits)	FOS + PHGG (biscuits)	Stool frequency and consistency; abdominal pain, intestinal bloating, and flatulence	Treatment stools more often described as formed soft, especially during first few days. Treatment ↑ mild and moderate abdominal pain and moderate flatulence and intestinal bloating. *n* = 2 reported severe or significant ↑ flatulence (and ↑ bloating in 1 of these participants)
Washington, 1998 (138)	Healthy adults in the United Kingdom (*n* = 8), 19–23 y	Randomized, 2-way crossover	Each study leg had 5-d period separated by 1-wk washout	10.5 g/d	None (lactulose)	Fybogel psyllium (lactulose)	Gastric emptying, small bowel and colonic transit	Psyllium slowed gastric emptying and colonic transit.^2^ Both treatment and control ↑ flatulence. Psyllium ↑ mild constipation in *n* = 2
Williams, 2004 (142)	Healthy adults in the United States (*n* = 48), 19–75 y, BMI 26.0 ± 0.4	Randomized, double blind, crossover	Two 24-h periods	5.5 g/d guar gum, 1.6 g/d alginate	None (crispy bar)	Guar gum + ISP Alginates alginate (crispy bar)	Questionnaire on frequency and intensity of nausea, abdominal cramping, distention, and flatulence for 24 h after treatment	No difference in tolerance symptoms

1 BMI is presented as kg/m^2^. FOS, fructooligosaccharides; ITF, inulin-type fructan; OF, oligofructose; PHGG, partially hydrolyzed guar gum.

2 Differences were statistically significant (*P* ≤ 0.05).

The inulin-type fructan mixtures ranged in dose from 9–16 g/d, with 9 g/d leading to mild flatulence, 15 g/d having mild to moderate or severe symptoms, and 16 g/d leading to moderate symptoms. Therefore, a therapeutic dose of 8–10 g/d inulin + OF to improve mineral absorption would lead to only mild flatulence. In addition, FOS + guar gum led to moderate symptoms, and psyllium + lactulose led to mild constipation. Additional studies are warranted to make recommendations for mixtures of inulin-type fructans and other NDCs.

## Discussion

Numerous studies demonstrate the health-promoting benefits of the consumption of NDCs by healthy adults. However, many trials testing health benefits do not report on the tolerance and functional effects of NDC consumption. Although information about bloating, flatulence, and stool frequency may not be primary outcomes, this information is critical to ensure translation of the findings. Indeed, we suggest that tolerance reporting in NDC studies (and other dietary trials) should become common practice, as the purpose of these studies is to translate knowledge for therapeutic purposes for patients and the well-being of consumers (13). Although a specific fiber dose may improve an aspect of human health, the consumer is unlikely to consume the dose if they cannot tolerate the gastrointestinal side effects. Therefore, explicitly measuring gastrointestinal effects, including both tolerance and functional effects, is essential in determining NDC tolerable intake doses to improve human health.

In addition, because of the considerable variation in types of NDCs, gastrointestinal effects need to be considered separately for each when making intake recommendations. These differing effects on tolerance and digestive function are caused by their unique physicochemical properties, including degree of solubility, viscosity, and fermentability for nonstarch polysaccharides, as well as by type, structural properties, and processing and modification for resistant starches (Figure 1). For example, soluble fibers with high viscosity slow gastric emptying, as observed with gel-forming guar gum (49). Another instance is fermentability, as demonstrated with inulin-type fructans, which can create flatulence and bloating. Furthermore, even among different inulin-type fructans, chain length can also affect fermentability, with shorter-chain fibers being fermented more rapidly by intestinal microorganisms compared with longer-chain fibers (65). In addition, although foods with inulin-type fructans and α-GOS are restricted on low-fermentation diets for individuals with irritable bowel syndrome, other fibers such as β-GOS are fermented by specific bacteria that do not produce as much gas, thereby decreasing discomfort and increasing tolerability (145). These individual properties also underscore why, although there are commonalities within fiber groups, it is important to also investigate individual fibers to determine appropriate doses. Moreover, combinations of fibers can act differently when coadministered than on their own. For instance, consuming inulin + psyllium decreased gas production in the colon of patients with irritable bowel syndrome compared with inulin alone (146). This highlights the importance of testing NDC combinations prior to incorporation into food products that would be available on the market.

We were not able to make recommendations for upper intake levels based on gastrointestinal effects for all of the currently FDA-approved dietary fibers. Although each of these approved fibers has evidence regarding benefits for varying aspects of health, many of them do not have sufficient studies reporting on tolerance. Nonstarch polysaccharide examples of these fibers are locust bean gum, glucomannan, hydroxypropylmethylcellulose, sugar cane fiber, mixed plant cell wall fibers, and lignin. Also, although we were able to review some studies reporting gastrointestinal effects of NDCs such as cellulose, lack of reporting on tolerance symptoms (e.g., flatulence, bloating) made it difficult to provide a tolerable intake recommendation. Because cellulose is an insoluble and nonfermentable fiber, reporting of gastrointestinal effects is often more related to functional stool characteristics (e.g., consistency) rather than tolerance symptoms such as bloating. This is the same with lignin and hydroxypropylmethylcellulose, which are also nonfermentable. However, NDCs such as locust bean gum and glucomannan are fermentable and therefore would be more likely to cause bloating and decreased tolerance.

In addition, the vehicle for NDC consumption (i.e., solid compared with liquid) should also be considered further. Solid vehicles include products such as bars, breads, pasta, and mixing into control meals, whereas liquid vehicles may include water, juice, soups, smoothies, or other beverages. For instance, although 1 study reported that guar gum was better tolerated using a solid vehicle (crispbread) rather than hydrated or semihydrated guar (41), more research is needed to confirm this finding. Overall, solid foods have a slower gastric emptying rate compared with liquids (147), thereby shifting the rate of fiber fermentation and the resulting gastrointestinal effects. Trials directly comparing vehicle forms are likewise warranted for all NDCs. Moreover, vehicle form could also affect the physiologic and health properties of the NDCs in addition to affecting tolerance. Depending on the food form, foods are stored and prepared differently before consumption. For instance, bread may be heated, whereas juice may be kept cool. However, in addition to food form, the product itself could change the preparation method—although soups and juices are both liquid vehicles, soup would be heated, whereas juice would likely be refrigerated. Variations in initial production and later preparation should all be considered when formulating a product with added NDC because heating and cooling can affect the bioavailability and structure of the NDCs (148). It is also important to test and report the characteristics of the NDC under the same conditions that it is consumed. For example, if a pasta dish containing the NDC is cooked, cooled, and then reheated prior to consumption, the same pattern should be repeated just before the product analysis.

In addition, the resistant starch (also classified as a dietary fiber) with the most evidence is soluble corn fiber. However, the FDA has recently approved other resistant starches as dietary fibers, such as cross-linked, phosphorylated resistant starch 4, because of other health benefits. Therefore, additional studies measuring tolerance symptoms and gastrointestinal effects should be conducted with different resistant starches to make consumption recommendations. In addition, because of the quickly expanding use and synthesis of different resistant starches, it is imperative that resistant starch type and processing steps be clearly reported when publishing. To make overarching recommendations, these resistant starches must be able to be compared with one another and classified into similar groupings. Consistent search terms must also be developed beyond “resistant starch” to enable researchers to find specific types of resistant starch, especially when different companies have different brand names for very similar resistant starch products. Moreover, it is also important to provide a complete characterization of all NDCs, including molecular weight, monosaccharides, linkages, and other properties and specifications (13).

Moreover, transparent reporting of NDC purity information must also become standard practice. Many studies use fibers that are not 100% pure fiber. For example, 1 GOS product was 72.5% pure (149) and 1 soluble corn fiber product was at a minimum 70% pure (150). Although many studies do report purity, others do not. This information must be made available to replicate studies, draw conclusions, and synthesize data appropriately.

Overall, it is important to examine the tolerable intake dose of each NDC classified as a dietary fiber to ensure that therapeutic doses meant to provide health benefits are not also creating undesirable gastrointestinal symptoms. Based on the studies reviewed herein, doses found to provide health benefits from the fibers β-glucan, alginate, psyllium husk, GOS, and soy fiber align with very favorable tolerance profiles. The fibers guar gum, inulin, FOS and OF, polydextrose, resistant maltodextrin/dextrin, and inulin + OF are also well tolerated at therapeutic doses with only mild or moderate gastrointestinal symptoms.

Last, although it was not the focus of the current review, studying tolerance and gastrointestinal effects of NDCs is also important in child and infant populations. Although there are increasing studies measuring these effects in infants because of growing interest in supplementing formula with prebiotics (e.g., FOS, GOS), studies are greatly lacking in young children and adolescents. We found 7 studies reporting tolerance in infants without gastrointestinal disease (151–157), but we found only 3 studies in adolescents (150, 158, 159) and 1 study in young children (160) (**Supplemental Table 2**). Overall, soluble corn fiber seems to be the most studied in terms of tolerance in children and combinations of FOS + GOS in infant formulas. Up to 20 g/d soluble corn fiber seems to be tolerated, with only mild symptoms in older children. However, more research must be conducted in this understudied population to make recommendations on different NDCs in children.

### Recommendations

**FIGURE 3:**
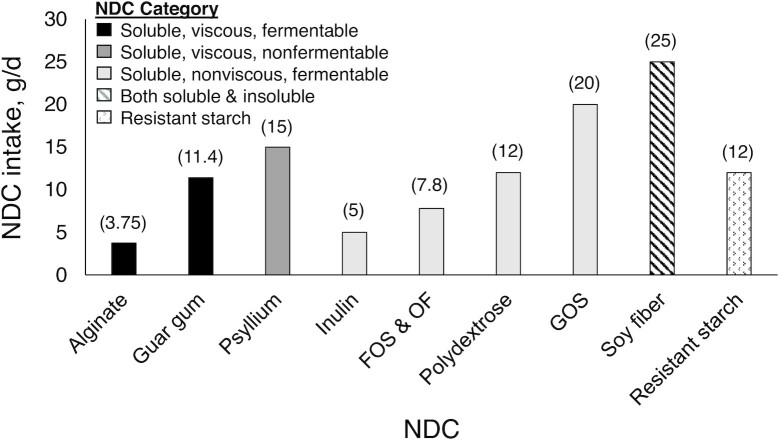
NDC tolerable intakes. Dose recommendations for tolerable intakes of NDCs grouped by category. FOS, fructooligosaccharides; GOS, galactooligosaccharides; NDC, nondigestible carbohydrate; OF, oligofructose.

For most adults to regularly consume a fiber product, it should not produce more than mild symptoms. Although consumers may tolerate slightly increased flatulence, they likely will not continue if the side effects are increased much further. Therefore, **Figure 3** and **Supplemental Table 1** display recommended tolerable intake dose thresholds for each discussed NDC that are likely to maintain symptoms at a level of mild or below for most adults based on the available evidence. These NDCs can be split into categories (as reviewed in Figure 1) based on solubility, viscosity, and fermentability.

An important consideration to point out with these recommendations is that individuals can adapt to increased doses of fiber consumption over time. Indeed, although there were a few single-meal challenges, most studies reviewed provided the fibers over a longer period of time of at least a week or more to examine the effects of daily consumption. Therefore, our dose recommendations are largely based on individuals who experienced a fiber adaptation period. Although adaptation can vary by habitual diet, fiber type, and dose, this period has been demonstrated to last for ∼2 weeks because of shifts in gastrointestinal microbiota and gas production (161).

In conclusion, although providing guidelines on total daily fiber consumption is important for the general population, it is also advisable to have tolerable intake recommendations for individual NDCs identified as dietary fibers, as well as for mixtures of these NDCs. This is because these fibers are increasingly used in food applications and to improve human health. Although individual fibers are listed in the ingredient lists of food and beverages, the individual fiber doses are not currently required to be included on the food label (only the composite amount). Thus, the suggested doses that are tolerable for intake by adults without gastrointestinal disease are likely to be primarily used by researchers designing clinical trials and companies formulating or reformulating food products. In these instances, we suggest reviewing and adapting the guidance on designing trials to assess tolerance to NDCs by Holscher et al. (13).

In summary, this review investigated gastrointestinal effects and tolerance of NDCs that meet the FDA definition of dietary fiber, recommended consumption doses ranging from 3.75–25 g/d of various NDCs, and highlighted the importance of developing standardized protocols to assess gastrointestinal tolerance and functional effects among dietary clinical trials.

## Supplementary Material

nmac094_Supplemental_File

## Notes

Publication fees were covered by the Institute for the Advancement of Food and Nutrition Sciences (IAFNS) Carbohydrate Committee. IAFNS is a nonprofit science organization that pools funding from industry and advances science through the in-kind and financial contributions from private and public sector members.

HDH has received grant funding from Danone, Tate & Lyle, General Mills, and speaking honoraria from Beneo and the Calorie Control Council. ARM has no conflicts of interest..

Supplemental Figures 1–2 are available from the “Supplementary data” link in the online posting of the article and from the same link in the online table of contents at https://academic.oup.com/advances/.

Abbreviations used: FOS, fructooligosaccharides; GOS, galactooligosaccharides; NDC, nondigestible carbohydrate; OF, oligofructose.
