# Supplementary material for: Gastrointestinal Effects and Tolerance of Nondigestible Carbohydrate Consumption
Source: Adv Nutr. 2022 Aug 30;13(6):2237–76. doi: 10.1093/advances/nmac094 (PMC9776669; doi:10.1093/advances/nmac094)
Supplement: nmac094_Supplemental_File [file nmac094_supplemental_file.pdf]

Supplemental Table 1. Tolerable intake dose recommendations for non-digestible carbohydrate mixtures

NDC	Dose Recommendation (g/d)
Soluble, viscous, fermentable	
Alginate	3.75
Guar gum	11.4
Soluble, viscous, nonfermentable	
Psyllium husk	15
Soluble, nonviscous, fermentable	
Inulin	5
FOS and OF	7.8
Polydextrose	12
GOS	20
Soluble and insoluble	
Soy fiber	25
Resistant starch	12

Abbreviations: NDC, non-digestible carbohydrate; FOS, fructooligosaccharides; OF, oligofructose; GOS, galactooligosaccharides

Supplemental Table 2. Clinical trials that studied non-digestible carbohydrate consumption in children and infants without gastrointestinal disease

Study	Population	Design	Duration	Dose	Control (vehicle)	Treatment (vehicle)	Assessment	Responses
Whisner, 2016 (158)	Healthy adolescent females in the United States (n=28), 11–14 y	3-phase, double-blind, crossover	4-wk doses, 3-d visits, 3-wk washouts	10, 20 g/d	Maltodextrin (muffin & fruit-flavored beverage)	Promitor SCF 85 (muffin & fruit-flavored beverage)	Weekly questionnaires to assess symptoms (flatulence, bloating, abdominal pain, diarrhea, stomach noises) from 0 (no symptoms) to 5 (severe)	20 g/d SCF ↑ mild flatulence & bloating compared to control*.
Whisner, 2014 (150)	Healthy adolescents in the United States (n=24), 12-15 y	Crossover	Two 3-wk periods	12 g/d	None (fruit snacks)	Promitor SCF 70 (fruit snacks)	Stomach noises, flatulence, bloating, & abdominal pain evaluated daily with questionnaire; symptom severity assessed daily using scale of 0 (none) to 10 (very severe)	No differences in symptom severity between treatments.
Whisner, 2013 (159)	Healthy adolescent females in the United States (n=31), 10-13 y	Randomized, double-blind, 3-period, crossover	13 wk (three 3-wk treatments, two 2-week washouts)	5, 10 g/d	None (smoothie drinks)	Vivinal GOS syrup, 59% pure (smoothie drinks)	Weekly phone survey with free-response answers & rankings from 0 (absent) to 5 (severe) for abdominal pain, bloating, flatulence, diarrhea, stool frequency & consistency	Mean abdominal pain, bloating, flatulence, & diarrhea scores were all below 1 (hardly any symptoms), with no differences between doses.
Lohner, 2018 (160)	Children in Hungary (n=219), 3-6 y	Parallel-group	24 wk	6 g/d	Maltodextrin (mixed in food or drink)	Orafti ITFs (mixed in food or drink)	Stool consistency	Treatment softened stool within normal range from ≥12 wk*.
Moro, 2002 (151)	Term infants in Italy (n=90)	Randomized, 3-way parallel-arm	28 d	0.4, 0.8 g/dL	Maltodextrin (formula)	FOS+GOS (formula)	Stool consistency rated 1 (watery) to 5 (hard) & frequency; consistency of each stool sample collected in 2 study days & mean obtained for each day; incidence of crying,	Stool frequency differed between 0.8 g/d FOS+GOS & control*. Dose-dependent influence on stool consistency.

							regurgitation, & vomiting	
Euler, 2005 (152)	Healthy term infants in the United States (n=72), 2-6 wk	Prospective, randomized, crossover (non-randomized human milk comparator group)	5 wk	1.5 or 3.0 g/L	None (human milk)	FOS (S-26 Gold formula)	Diary recording stool frequency, size, consistency, & color completed during 24 h before each weekly visit; stool consistency rated 1 (hard) to 5 (watery); stool frequency reported as number of stools in past 24 h; parent/guardian questioned on adverse events at each visit	3.0 g/L FOS softened stool compared to 1.5 g/L FOS*.
Moro, 2006 (153)	Infants in Italy (treatment: n=102, control: n=104) at risk of atopy	Double-blind, randomized, placebo-controlled, parallel-group	6 months	0.8 g/100 ml	Maltodextrin (formula)	GOS+FOS (formula)	Incidence of crying, regurgitation, & vomiting (3-point scale) recorded in parent interview; stool characteristics of consistency (5-point scale) & frequency	Treatment significantly impacted stool frequency & consistency*. FOS+GOS ↓ regurgitation & crying*.
Rodriguez-Herrera, 2019 (154)	Infants in Italy & Spain (n=200), ≤28 d	Multi-center, prospective, double-blind, randomized, controlled	≤28 days-17 wk of age	0.8 g/100 ml	None (non-fermented formula), none (human milk, breast-fed)	GOS+FOS (fermented formula)	Gastrointestinal symptoms, stool characteristics	GOS+FOS softened stool consistency compared to control, with values closer to breastfed*. GOS+FOS ↓ colic compared to control*.
Ziegler, 2007 (155)	Healthy infants in the United States (n=226)	3-way parallel-arm	120 d	4, 8 g/L	None (formula)	PDX+GOS (formula)	24-hour tolerance recall at 30, 60, 90, & 120 d of age; adverse events recorded throughout study	Treatments caused looser stools compared to control*. 8 g/L ↑ frequency compared to control & 4 g/L at 30 d*. 4 g/L differed from control in diarrhea & 8 g/L differed from control in irritability*.

Moore, 2003 (156)	Healthy infants in the United States (n=56), 16-46 wk	Parallel-arm	28 d	0.75 g/serving (mean 0.74 g/d, max 3 g/d FOS)	Maltodextrin (cereal)	FOS (Nestle Carnation Premium Baby Cereal)	Gastrointestinal tolerance assessed by daily parental reporting (stool patterns, tolerance symptoms)	FOS softened stool consistency & ↑ frequency* compared to control. FOS well-tolerated at ≤3 g/d.
Waligora-Dupriet, 2007 (157)	20 healthy infants & toddlers in France (n=20), 7-19 months	Double-blind, placebo-controlled	8-d observation, 21-d treatment, 15-d post-treatment	2 g/d	Maltodextrin (cereal or drinks)	Orafti Beneo P95 OF (cereal or drinks)	Intestinal tolerance & well-being	OF ↓ flatulence, diarrhea, & vomiting compared to control*.

Abbreviations: FOS, fructooligosaccharides; GOS, galactooligosaccharides; OF, oligofructose; PDX, polydextrose; SCF, soluble corn fiber

* Differences were statistically significant ($P \le 0.05$).
